# Exploration of the association between the single-nucleotide polymorphism of co-stimulatory system and rheumatoid arthritis

**DOI:** 10.3389/fimmu.2023.1123832

**Published:** 2023-06-29

**Authors:** Ding-Ping Chen, Ying-Hao Wen, Wei-Tzu Lin, Fang-Ping Hsu, Kuang-Hui Yu

**Affiliations:** ^1^ Department of Laboratory Medicine, Linkou Chang Gung Memorial Hospital, Taoyuan, Taiwan; ^2^ Department of Medical Biotechnology and Laboratory Science, College of Medicine, Chang Gung University, Taoyuan, Taiwan; ^3^ School of Medicine, National Tsing Hua University, Hsinchu, Taiwan; ^4^ School of Medicine, Chang Gung University, Taoyuan, Taiwan; ^5^ Graduate Institute of Clinical Medical Sciences, College of Medicine, Chang Gung University, Taoyuan, Taiwan; ^6^ Division of Rheumatology, Allergy, and Immunology, Linkou Chang Gung University and Memorial Hospital, Taoyuan, Taiwan

**Keywords:** rheumatoid arthritis (RA), co-stimulatory system, single nucleotide polymorphism (SNP), autoimmune disease (AD), association

## Abstract

**Introduction:**

The human leukocyte antigen (HLA) has been linked to the majority of autoimmune diseases (ADs). However, non-HLA genes may be risk factors for ADs. A number of genes encoding proteins involved in regulating T-cell and B-cell function have been identified as rheumatoid arthritis (RA) susceptibility genes.

**Methods:**

In this study, we investigated the association between RA and single-nucleotide polymorphisms (SNPs) of co-stimulatory or co-inhibitory molecules in 124 RA cases and 100 healthy controls without immune-related diseases [including tumor necrosis factor superfamily member 4 (TNFSF4), CD28, cytotoxic T-lymphocyte–associated protein 4 (CTLA4), and programmed cell death protein 1 (PDCD1)].

**Results:**

The results showed that there were 13 SNPs associated with RA, including rs181758110 of TNFSF4 (CC vs. CT, *p* = 0.038); rs3181096 of CD28 (TT vs. CC + CT, *p* = 0.035; CC vs. TT, *p* = 0.047); rs11571315 (TT vs. CT, *p* = 0.045), rs733618 (CC vs. TT + CT, *p* = 0.043), rs4553808 (AA vs. AG vs. GG, *p* = 0.035), rs11571316 (GG vs. AG vs. AA, *p* = 0.048; GG vs. AG + AA, *p* = 0.026; GG vs. AG, *p* = 0.014), rs16840252 (CC vs. CT vs. TT, *p* = 0.007; CC vs. CT, *p* = 0.011), rs5742909 (CC vs. CT vs. TT, *p* = 0.040), and rs11571319 of CTLA4 (GG vs. AG vs. AA, *p* < 0.001; GG vs. AG + AA, *p* = 0.048; AA vs. GG + AG, *p* = 0.001; GG vs. AA, *p* = 0.008; GG vs. AG, *p* ≤ 0.001); and rs10204525 (TT vs. CT + CC, *p* = 0.024; TT vs. CT, *p* = 0.021), rs2227982 (AA vs. GG, *p* = 0.047), rs36084323 (TT vs. CT vs. CC, *p* = 0.022; TT vs. CT + CC, *p* = 0.013; CC vs. TT + CT, *p* = 0.048; TT vs. CC, *p* = 0.008), and rs5839828 of PDCD1 (DEL vs. DEL/G vs. GG, *p* = 0.014; DEL vs. DEL/G + GG, *p* = 0.014; GG vs. DEL + DEL/G, *p* = 0.025; DEL vs. GG, *p* = 0.007).

**Discussion:**

Consequently, these SNPs may play an important role in immune regulation, and further research into the role of these SNPs of immune regulatory genes in the pathogenesis of RA is required.

## Introduction

Rheumatoid arthritis (RA) is a chronic inflammatory autoimmune disease (AD) in which the immune system attacks the joints as a result of abnormal autoimmunity. The prevalence rate of RA ranges from 0.24% to 1% of the global population (1). Most patients’ hands, feet, joints, and jaw will be affected, potentially leading to joint deformation. Aside from joints, the skin, eyes, and blood vessels can also be affected.

RA is a genetic AD identified through previous family twin studies (2). Furthermore, Kurkó et al. (3) stated that genetics, environmental factors, and autoimmunity were the three major factors causing RA pathogenesis, with genetics accounting for 60% of the total. Although the pathogenesis of RA is still unknown, numerous studies have shown that the loss of immune tolerance caused by autoreactive T-cell overactivation is the primary cause of AD (4, 5). Human leukocyte antigen (HLA) genes have been found to have a strong correlation with AD (6). However, other genes found outside the HLA region may be risk factors for AD. Studies have shown that T-cell activation is strictly regulated by signals from co-stimulatory and co-inhibitory molecules (7). Therefore, we focus on the SNPs of the co-stimulatory system, including tumor necrosis factor superfamily member 4 (TNFSF4 and OX40L), CD28, cytotoxic T-lymphocyte–associated protein 4 (CTLA4 and CD152), and programmed cell death protein 1 (PDCD1 and CD279).

Both TNFSF4 and CD28 stimulate T-cell activation. TNFSF4 and its receptor, OX40, interact to promote T-cell survival, activation, and differentiation (8). TNFSF4 may also influence immune tolerance by activating OX40 (8). Immune tolerance loss is well known to cause autoimmune disorders (9). CD28 is an important co-stimulatory molecule. The CD28 signal generated by the CD28 and CD80/CD86 interaction plays a critical role in T-cell activation and differentiation, and it has been demonstrated that the CD28 signal plays a key role in regulating immune tolerance and autoimmunity in animal models demonstrated in an animal model (10). Furthermore, CTLA4 and PDCD1 inhibit T-cell activation by blocking CD28-mediated upregulation (11). These co-stimulatory and co-inhibitory molecules have also been used as immunotherapy targets (12–15), indicating that they play an important role in immune regulation and disease resolution.

A single-nucleotide polymorphism (SNP) is a common genetic variation that is often used as a genetic marker in the study of genetic diseases. Moreover, genetic polymorphisms can be used as prognostic markers for the prognosis of RA. However, SNP combinations in genomes differ from person to person, particularly among ethnic groups. Although foreign teams have investigated the association between ADs and immune regulatory genes, it is still unclear whether the susceptibility of SNPs applies to Taiwanese people. Therefore, studying disease SNPs for specific ethnic groups remains worthwhile.

## Materials and methods

### Study subjects

In this study, 124 patients with RA and 100 subjects without immune abnormalities were recruited. All patients and healthy subjects were asked to sign informed consent forms before we collected their peripheral blood samples, and all procedures were performed in accordance with the applicable guidelines and regulations. The study was approved by the Institutional Review Board (IRB) of Chang Gung Memorial Hospital (CGMH; IRB no. 202002097B0 and 202102018B0C601). The inclusion criteria of RA were based on the RA classification criteria (16). In addition, the healthy controls were recruited from the general population: those without ADs and immune abnormalities or those using immunosuppressive drugs.

### Polymerase chain reaction and SNP analysis

The genomic DNA was first extracted from peripheral blood samples using the QIAamp DNA Blood Mini Kit (Qiagen, Valencia, CA). The concentration and purity of DNA were then determined using a NanoDrop ND-1000 UV–Vis Spectrophotometer (Thermo Fisher Scientific Inc., Waltham, MA) in preparartion for the subsequent PCR. This study focused on the TNFSF4 gene on chromosome 1 and the CTLA4, CD28, and PDCD1 genes on chromosome 2. Because ADs can result from abnormal co-stimulatory and co-inhibitory molecule expression levels (17) and SNP variation in the promoter region can affect gene expression (18), the promoter region of genes was included. In addition, previously studied hotspots, such as rs3087243 at the 3′ untranslated region (3′UTR) of CTLA4 gene, rs6705653 and rs41386349 at the exon 4 of PDCD1 gene, rs2227981 at the exon 5 of PDCD1 gene, and re10204525 at the 3′UTR of PDCD1 gene, were included. We obtained gene polymorphism data related to 37 SNPs of the above genes from the SNP database of NCBI and designed eight pairs of primers for detecting these genes (**Table 1**) to amplify genomic DNA fragments containing these 37 SNPs (**Table 2**). Because of poor quality genomic DNA and PCR failure, not all samples had complete SNP data.

**Table 1:** The pairs of primers for amplifying the specific region of TNFSF4, CD28, CTLA4, and PDCD1 gene.

Gene	Genomic region	Primer sequence	PCR product (bp)
TNFSF4	Promoter and exon 1	F: 5′-GGCTTGGAGTCTATGATATTGTGCC-3′ R: 5′-GAAGGGCGTTTAACCACACTTTACG-3′	1,725
CD28	Promoter and exon 1	F: 5′- GGGTGGTAAGAATGTGGATGAATC-3′ R: 5′-CAAGGCATCCTGACTGCAGCA-3′	1,961
	intron 3	F: 5′-CGGATGCAGTTTAGGGTCTAGATT-3′ R: 5′-GATCAAGCCAACATTGTCCATTGG-3′	
CTLA4	Promoter	F: 5′-GGCAACAGAGACCCCACCGTT-3′ R: 5′-GAGGACCTTCCTTAAATCTGGAGAG-3′	1,233
	Promoter and exon 1	F: 5′-CTCTCCAGATTTAAGGAAGGTCCTC-3′ R: 5′-GGAATACAGAGCCAGCCAAGCC-3′	1,169
	Exon 4 and 3′UTR	F: 5′-GCTTGGAAACTGGATGAGGTCATAGC-3′ R: 5′-AGAGGAAGAGACACAGACAGAGTTGC-3′	1,204
PDCD1	Promoter and exon 1	F: 5′-ACCCACACAGCCTCACATCTCT-3′ R: 5′-AAACTGAGGGTGGAAGGTCCCT-3′	1,778
	Exon 4, intron 4, and exon 5	F: 5′-TGGTGACCCCAAGTGTGTTTCTC-3′ R: 5′-GAGGAATTTTTCACCGGAGGGC-3′	2,234

F, forward primer; R, reverse primer.

**Table 2:** The 37 target SNPs located in TNFSF4, CD28, CTLA4, and PDCD1 gene for analysis.

Gene	Genomic region	SNP under analysis				
TNFSF4	Promoter	rs181758110	rs45454293	rs1234314		
						
CD28	Promoter	rs1879877	rs3181096	rs3181097	rs3181098	rs28718975
		rs28688913	rs28541784	rs201801072	rs200353921	rs56228674
		rs3116496	rs1290180288			
						
CTLA4	Promoter	rs11571315	rs733618	rs4553808	rs11571316	rs62182595
		rs16840252	rs945677329	rs5742909		
	Exon 1	rs231775				
	Exon 4	rs56217811	rs980967681	rs55696217		
	3′UTR	rs3087243	rs11571319			
						
PDCD1	Promoter	rs36084323	rs5839828			
	Intron 4	rs6705653	rs41386349			
	Exon 5-3′UTR	rs10204525	rs56029561	rs2227981	rs2227982	

### Statistical analysis

First, the Hardy–Weinberg equilibrium (HWE) was used to examine the allele frequencies of each SNP in the control group. For statistical analysis, the chi-square test and Fisher’s exact test were performed using SPSS17.0 software. The allele with the highest frequency in the population recruited for this study was designated as major allele “A” and the other as minor allele “a”. In genotype analysis, the homozygous of major allele (AA) was defined as reference, and the homozygous model (AA vs. aa), heterozygote model (AA vs. Aa), additive model (AA vs. Aa vs. aa), dominant model (AA vs. Aa + aa), and recessive model (AA + Aa vs. aa) were evaluated. The significance level was set at α = 0.05, and the odds ratio (OR) with 95% confidence interval (95% CI) was provided. D was used to estimate linkage disequilibrium (LD) by comparing the observed and expected frequency of a haplotype involved in alleles from different loci. Gabriel et al. (19) defined a haplotype block and ecluded haplotypes with a frequency of less than 1%. The figure of LD was generated using Haploview 4.2 (https://www.broadinstitute.org/haploview/haploview).

## Results

In this study, 124 patients with RA were recruited, with 101 (81%) women and 23 men (19%). Their onset age was 45.69 ± 1.22 years old. Moreover, 100 people with no immune abnormalities were included in this study, with 74 (74%) women and 26 men (26%), and their average age was 32.1 ± 8.9 years old. The clinical characteristics of all patients are summarized in **Table 3**.

**Table 3:** The clinical characteristics of participants recruited in this study.

Variable	Cases (n = 124)	Controls (n = 100)
Female: male	101:23	74:26
Age (mean ± SD; years old)		32.1 ± 8.9
Age at onset (mean ± SD; years old)	45.69 ± 1.22	
Disease duration (mean ± SD; years)	14.99 ± 0.81	
RF-positive (n; %)	92 (74%)	
ACCP-positive	20 (16%)	
CRP-positive	75 (60%)	
DAS28 (mean ± SD)	3.92 ± 0.13	
ESR (mean ± SD; mm/h)	24.29 ± 2.07	
GH (mean ± SD)	37.13 ± 2.06	

RF, rheumatoid factor; ACCP, anti-cyclic citrullinated peptide antibody; CRP, C-reactive protein; ESR, erythrocyte sedimentation rate; DAS28 is referred to the disease activity of RA; GH, general health.

### Hardy–Weinberg equilibrium

HWE equilibrium was used to determine whether the control group included could accurately represent the entire population. The findings revealed that rs28718975, rs28541784, rs201801072, rs200353921, and rs1290180288 of CD28 gene and rs945677329, rs56217811, rs980967681, and rs55696217 of CTLA4 gene deviated from HWE. Because subsequent analyses of these SNPs had low confidence, they were not analyzed and discussed (**Table 4**).

**Table 4:** The HWE analysis of each SNP from the control group.

Gene	Genomic region	SNP under analysis	HWE
TNFSF4	Promoter	rs181758110	0.98
		rs45454293	0.95
		rs1234314	0.32
CD28	Promoter	rs1879877	0.86
		rs3181096	0.20
		rs3181097	0.96
		rs3181098	0.16
		rs28718975	0.03*
		rs28688913	0.50
		rs28541784	0.01*
		rs201801072	0.00*
		rs200353921	0.00*
		rs56228674	0.82
		rs3116496	0.67
		rs1290180288	NA*
CTLA4	Promoter	rs11571315	0.81
		rs733618	0.89
		rs4553808	0.43
		rs11571316	1.00
		rs62182595	0.95
		rs16840252	0.36
		rs945677329	NA*
		rs5742909	0.24
	Exon 1	rs231775	0.88
	Exon 4	rs56217811	NA*
		rs980967681	0.03*
		rs55696217	NA*
	3′UTR	rs3087243	1.00
		rs11571319	0.17
PDCD1	Exon 5	rs10204525	0.99
		rs56029561	0.94
		rs2227981	0.31
		rs2227982	0.98
	Intron 4	rs6705653	0.56
		rs41386349	0.49
	Promoter	rs36084323	0.97
		rs5839828	0.65

Asterisk (*) indicates that the SNP was deviated from HWE (p < 0.05). NA, not applicable.

### The association between RA and SNPs

The genotype frequencies of SNPs located in four genes—TNFSF4, CD28, CTLA4, and PDCD1—were compared between patients with RA and healthy controls in this study. On the basis of the results, 13 SNPs were associated with RA. One was in the TNFSF4 gene, one in the CD28 gene, seven in the CTLA4 gene, and four in the PDCD1 gene (**Table 5**). The raw data are shown in **Supplementary Tables 1–4**.

**Table 5:** The SNPs associated with RA.

SNP	Gene position	No. of patients (%)			Model	Model	Logistic regression p	OR (95% CI)	
rs181758110	173208023	GG	AG	AA	Additive	GG vs. AG vs. AA	0.038*	NA	
control	TNFSF4	96	4	0	Dominant	GG vs. AG + AA	0.038*	NA	
		44%	100%	0%	Recessive	GG + AG vs. AA	NA	NA	
RA		124	0	0	Homozygous	GG vs. AA	NA	NA	
		56%	0%	0%	Heterozygous	GG vs. AG	0.038*	NA	
rs3181096	203705369	CC	CT	TT	Additive	CC vs. CT vs. TT	0.101	NA	
control	CD28	58	31	10	Dominant	CC vs. CT + TT	0.774	0.924	(0.540–1.580)
		44%	41%	71%	Recessive	CC + CT vs. TT	0.035*	0.297	(0.090–0.980)
RA		75	45	4	Homozygous	CC vs. TT	0.047*	0.309	(0.920–1.040)
		56%	59%	29%	Heterozygous	CC vs. CT	0.692	1.123	(0.630–1.990)
rs11571315	203866178	TT	CT	CC	Additive	TT vs. CT vs.CC	0.117	NA	
control	CTLA4	47	41	12	Dominant	TT vs. CT + CC	0.105	0.643	(0.380–1.100)
		41%	55%	41%	Recessive	TT + CT vs. CC	0.619	1.222	(0.550–2.700)
RA		69	33	17	Homozygous	TT vs. CC	0.933	0.965	(0.420–2.200)
		59%	45%	59%	Heterozygous	TT vs. CT	0.045*	0.548	(0.300–0.990)
rs733618	203866221	TT	CT	CC	Additive	TT vs. CT vs.CC	0.110	NA	
control	CTLA4	36	46	18	Dominant	TT vs. CT + CC	0.742	1.098	(0.630–1.910)
		47%	51%	33%	Recessive	TT + CT vs. CC	0.043*	1.929	(1.020–3.670)
RA		41	44	36	Homozygous	TT vs. CC	0.125	1.756	(0.850–3.610)
		53%	49%	67%	Heterozygous	TT vs. CT	0.574	0.840	(0.460–1.550)
rs4553808	203866282	AA	AG	GG	Additive	AA vs.AG vs. GG	0.035*	NA	
control	CTLA4	77	23	0	Dominant	AA vs. AG + GG	0.205	0.650	(0.330–1.270)
		43%	59%	0%	Recessive	AA + AG vs. GG	0.130	NA	
RA		103	16	4	Homozygous	AA vs. GG	0.141	NA	
		57%	41%	100%	Heterozygous	AA vs. AG	0.066	0.520	(0.260–1.050)
rs11571316	203866366	GG	AG	AA	Additive	GG vs. AG vs. AA	0.048*	NA	
control	CTLA4	60	35	5	Dominant	GG vs. AG + AA	0.026*	0.527	(0.300–0.930)
		40%	58%	42%	Recessive	GG + AG vs. AA	0.820	1.147	(0.350–3.730)
RA		91	25	7	Homozygous	GG vs. AA	1.000	0.923	(0.280–3.040)
		60%	42%	58%	Heterozygous	GG vs. AG	0.014*	0.471	(0.260–0.870)
rs16840252	203866796	CC	CT	TT	Additive	CC vs. CT vs. TT	0.007*	NA	
control	CTLA4	75	25	0	Dominant	CC vs. CT + TT	0.051	0.514	(0.260–1.010)
		42%	64%	0%	Recessive	CC + CT vs. TT	0.130	NA	
RA		105	14	4	Homozygous	CC vs. TT	0.147	NA	
		58%	36%	100%	Heterozygous	CC vs. CT	0.011*	0.400	(0.200–0.820)
rs5742909	203867624	CC	CT	TT	Additive	CC vs. CT vs. TT	0.040*	NA	
control	CTLA4	71	29	0	Dominant	CC vs. CT + TT	0.340	0.747	(0.410–1.360)
		43%	55%	0%	Recessive	CC + CT vs. TT	0.067	NA	
RA		95	24	5	Homozygous	CC vs. TT	0.077	NA	
		57%	45%	100%	Heterozygous	CC vs. CT	0.128	0.619	(0.330–1.150)
rs11571319	203874215	GG	AG	AA	Additive	GG vs. AG vs. AA	<0.001*	NA	
control	CTLA4	61	38	1	Dominant	GG vs. AG + AA	0.048*	0.567	(0.320–1.000)
		40%	68%	6%	Recessive	GG + AG vs. AA	0.001*	13.624	(1.770–105.03)
RA		91	18	15	Homozygous	GG vs. AA	0.008*	10.06	(1.290–78.11)
		60%	32%	94%	Heterozygous	GG vs. AG	<0.001*	0.318	(0.170–0.610)
rs10204525	241850169	TT	CT	CC	Additive	TT vs. CT vs.CC	0.069	NA	
control	PDCD1	65	31	4	Dominant	TT vs. CT + CC	0.024*	1.857	(1.080–3.190)
		51%	35%	44%	Recessive	TT + CT vs. CC	1.000	1.008	(0.264–3.859)
RA		62	57	5	Homozygous	TT vs. CC	0.743	1.310	(0.340–5.110)
		49%	65%	56%	Heterozygous	TT vs. CT	0.021*	1.928	(1.100–3.370)
rs2227982	241851281	AA	AG	GG	Additive	AA vs.AG vs. GG	0.139	NA	
control	PDCD1	38	46	15	Dominant	AA vs. AG + GG	0.138	1.531	(0.870–2.690)
		52%	45%	33%	Recessive	AA + AG vs. GG	0.078	1.846	(0.930–3.670)
RA		35	56	30	Homozygous	AA vs. GG	0.047*	2.171	(1.000–4.700)
		48%	55%	67%	Heterozygous	AA vs. AG	0.364	1.322	(0.720–2.410)
rs36084323	241859444	TT	CT	CC	Additive	TT vs. CT vs.CC	0.022*	NA	
control	PDCD1	40	43	13	Dominant	TT vs. CT + CC	0.013*	2.054	(1.160–3.640)
		56%	41%	30%	Recessive	TT + CT vs. CC	0.048*	2.038	(1.000–4.160)
RA		32	62	30	Homozygous	TT vs. CC	0.008*	2.885	(1.300–6.420)
		44%	59%	70%	Heterozygous	TT vs. CT	0.056	1.802	(0.980–3.300)
rs5839828	241859601	DEL	DEL/G	GG	Additive	DEL vs. DEL/G vs.GG	0.014*	NA	
control	PDCD1	50	41	5	Dominant	DEL vs. DEL/G + GG	0.014*	1.976	(1.150–3.400)
		53%	40%	22%	Recessive	DEL + DEL/G vs.GG	0.025*	3.091	(1.100–8.650)
RA		44	62	18	Homozygous	DEL vs.GG	0.007*	4.091	(1.400–11.93)
		47%	60%	78%	Heterozygous	DEL vs. DEL/G	0.060	1.718	(0.980–3.030)

Additive, AA vs. Aa vs. aa; dominant, AA vs. Aa + aa; recessive, AA + Aa vs. aa; homozygous, AA vs. aa; heterozygous, AA vs. Aa, where the frequency of A-allele is major in the population, and a-allele is minor. *, <0.05; NA, not applicable.

The genotype frequency of rs181758110 in the TNFSF4 gene differed significantly between RA cases and healthy controls (CC vs. CT, p = 0.038). In rs181758110, all RA cases had the CC genotype. rs3181096 in the CD28 gene was associated with RA in the homozygous model (CC vs. TT, p = 0.047, OR = 0.309, 95% CI = 0.92–1.04) and recessive model (CC + CT vs. TT, p = 0.035, OR = 0.297, 95% CI = 0.09–0.98). This meant that people with TT genotype were less likely to develop RA than people with the CC genotype or at least one C allele (CC and CT). Thus, the C allele would be a risk allele for RA.

Only one of the seven significant SNPs in the CTLA4 gene, rs11571319, was in the 3′UTR, whereas the other six SNPs were in the promoter region. On the basis of the heterozygous model, rs11571315 was associated with RA (TT vs. CT, p = 0.045, OR = 0.548, 95% CI = 0.30–0.99). Subjects with the CT genotype in rs11571315 had a lower risk of developing RA. The rs733618 was associated with RA in a recessive model (TT + CT vs. CC, p = 0.043, OR = 1.929, 95% CI = 1.02–3.67), which meant that subjects carrying the CC genotype had a 1.929 times higher risk of developing RA than subjects carrying at least one T allele (CT and TT). The genotype frequency of rs4553808 was associated with RA based on the additive model (AA vs. AG vs. GG, p = 0.035). The rs11571316 was associated with RA based on the additive model (GG vs. AG vs. AA, p = 0.048), heterozygous model (GG vs. AG, p = 0.014, OR = 0.471, 95% CI = 0.26–0.87), and dominant model (GG vs. AG + AA, p = 0.026, OR = 0.527, 95% CI = 0.30–0.93). The rs16840252 was associated with RA based on the additive model (CC vs. CT vs. TT, p = 0.007) and heterozygous model (CC vs. CT, p = 0.011, OR = 0.400, 95% CI = 0.20–0.82). The genotype frequency of rs5742909 was associated with RA based on the additive model (CC vs. CT vs. TT, p = 0.040). rs11571319 in the 3′UTR was associated with RA based on all the analysis model. The genotypes of patients with RA and healthy controls differed significantly (GG vs. AG vs. AA, p < 0.001). In comparison with GG, subjects with AG had a lower risk of developing RA (OR = 0.318, 95% CI = 0.17–0.61, p < 0.001), whereas subjects with AA had a higher risk of developing RA (OR = 10.06, 95% CI = 1.29–78.11, p = 0.008). In addition, when compared with AG + GG, patients with AA had a higher risk of developing RA (OR = 13.624, 95% CI = 1.77–105.03, p = 0.001).

Four SNPs in the PDCD1 gene were associated with RA. rs10204525 was associated with RA based on the heterozygous model (TT vs. CT, OR = 1.928, 95% CI = 1.10–3.37, p = 0.021) and dominant model (TT vs. CT + CC, OR = 1.857, 95% CI = 1.08–3.19, p = 0.024). In comparison with the AA genotype, subjects with GG at rs2227982 had a 2.171 times increased risk of developing RA (95% CI = 1.00–4.70, p = 0.047). The genotype frequencies of rs36084323 were significantly different between RA cases and healthy controls based on the additive model (TT vs. CT vs. CC, p = 0.022), dominant model (TT vs. CT + CC, OR = 2.054, 95% CI = 1.16–3.64, p = 0.013), recessive model (TT + CT vs. CC, OR = 2.038, 95% CI = 1.00–4.16, p = 0.048), and homozygous model (TT vs. CC, OR = 2.885, 95% CI = 1.30–6.42, p = 0.008). Furthermore, the genotype frequencies of rs5839828 were significantly different between RA cases and healthy controls based on the additive model (DEL vs. DEL/G vs. GG, p = 0.014), dominant model (DEL vs. DEL/G + GG, OR = 1.976, 95% CI = 1.15–3.40, p = 0.014), recessive model (DEL + DEL/G vs. GG, OR = 3.091, 95% CI = 1.10–8.56, p = 0.025), and homozygous model (DEL vs. GG, OR = 4.091, 95% CI = 1.40–11.93, p = 0.007).

### Linkage disequilibrium and haplotype analysis

RA may be associated with a specific haplotype because of the differences in prevalence and susceptible SNPs in different populations. Therefore, the haplotype analysis was performed after the SNP analysis.

**Figure 1:**
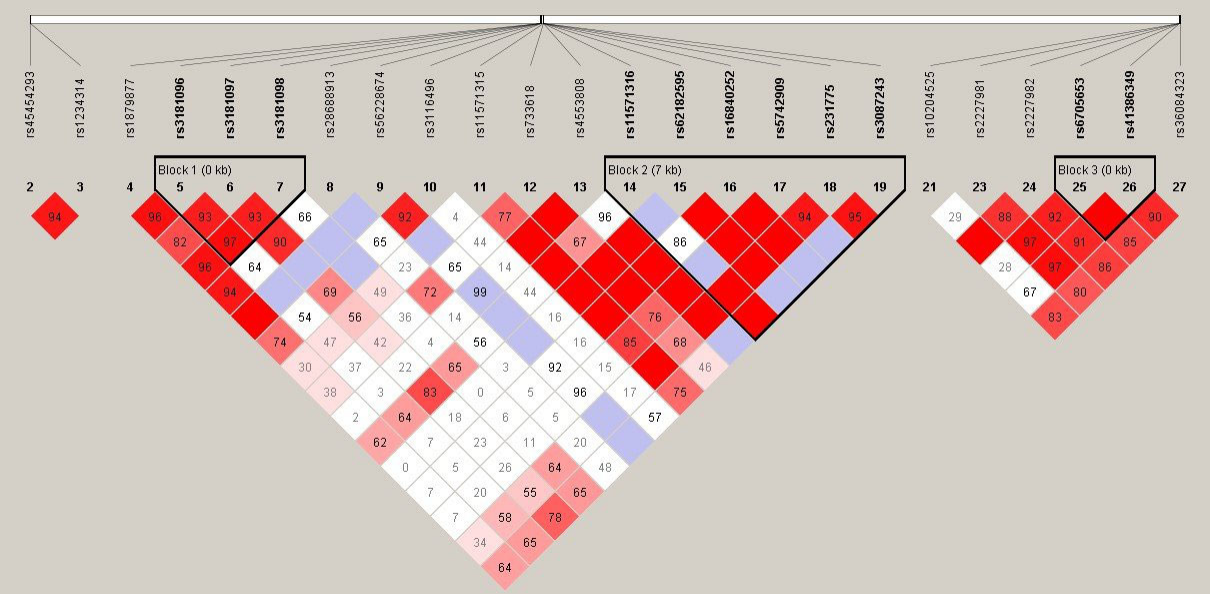
Linkage disequilibrium (LD) plot of TNFSF4, CD28, CTLA4, and PDCD1 gene. There was each one haplotype bolck in CD28, CTLA4, and PDCD1 gene. The CD28 haplotype contained rs3181096, rs3181097, and rs3181098. The CTLA4 haplotype contained rs11571316, rs62182595, rs16840252, rs5742909, rs231775, and rs3087243. The PDCD1 haplotype contained rs6705653and rs41386349.

In LD analysis, the red color in the box indicates that two SNPs have strong linkage, whereas the less the linkage, the closer to the white color of the box, and the light purple indicates no linkage. In addition, the high LD region is referred to as a haplotype block. According to **Figure 1**, there was one haplotype in the CD28 gene, composed of rs3181096, rs3181097, and rs3181098; one haplotype in the CTLA4 gene, composed of rs11571316, rs62182595, rs16840252, rs5742909, rs231775, and rs3087243; and one haplotype in the PDCD1 gene, composed of rs6705653 and rs41386349. After excluding the haplotypes with frequency less than 1%, it was found that 10 different types of haplotypes were statistically associated with RA (GATCAG—OR = 0.415, 95% CI = 0.197–0.874, p = 0.018; GATCGG and GATTGG—OR = 0.362, 95% CI = 0.148–0.885, p = 0.022; GGCCAG—OR = 0.547, 95% CI = 0.320–0.936, p = 0.027; GGCCGG—OR = 0.468, 95% CI = 0.224–0.976, p = 0.040; GGTCAG and GGTTAG—OR = 0.415, 95% CI = 0.197–0.874, p = 0.018; GGTTGG—OR = 0.337, 95% CI = 0.139–0.817, p = 0.013; AGCCAG—OR = 0.531, 95% CI = 0.285–0.991, p = 0.045; AGCCGG—OR = 0.416, 95% CI = 0.215–0.805, p = 0.008; **Table 6**).

**Table 6:** The haplotypes associated with RA.

GVHD	Haplotypes	Freq. cases	Freq. controls	OR	95% CI	*p*-value
CTLA4	GATCAG	0.105	0.220	0.415	(0.197–0.874)	0.018
	GATCGG	0.065	0.160	0.362	(0.148–0.885)	0.022
	GATTGG	0.065	0.160	0.362	(0.148–0.885)	0.022
	GGCCAG	0.363	0.510	0.547	(0.320–0.936)	0.027
	GGCCGG	0.774	0.880	0.468	(0.224–0.976)	0.040
	GGTCAG	0.105	0.220	0.415	(0.197–0.874)	0.018
	GGTTAG	0.105	0.220	0.415	(0.197–0.874)	0.018
	GGTTGG	0.065	0.170	0.337	(0.139–0.817)	0.013
	AGCCAG	0.185	0.300	0.531	(0.285–0.991)	0.045
	AGCCGG	0.145	0.290	0.416	(0.215–0.805)	0.008

Haplotype contained rs11571316A/G, rs62182595A/G, rs16840252C/T, rs5742909C/T, rs231775A/G, and rs3087243A/G.

Freq., frequency; OR, odds ratio; CI, confidence interval.

### The association between antibodies and SNPs

In addition to investigating the association between SNPs and the development of RA, the association between SNPs and immunological parameters was also been explored. There were three SNPs associated with rheumatoid factor (RF), one SNP associated with anti-cyclic citrullinated peptide antibody (ACCP), and eight SNPs associated with C-reactive protein (CRP) (**Table 7**). The raw data are shown in **Supplementary Tables 5–7**.

**Table 7:** The SNPs associated with the presence of RF, ACCP, and CRP.

SNP	Gene position	No. of patients (%)			Model		Logistic regression p	OR (95% CI)	
rs11571315	203866178	TT	CT	CC	Additive	TT vs.TC vs.CC	0.070	NA	
RF+	CTLA4	53	27	9	Dominant	TT vs. TC + CC	0.551	0.776	(0.338–1.785)
		77%	82%	53%	Recessive	TT + TC vs. CC	0.035*	0.309	(0.107–0.896)
RF−		16	6	8	Homozygous	TT vs. CC	0.070	0.340	(0.113–1.025)
		23%	18%	47%	Heterozygous	TT vs. TC	0.565	1.358	(0.477–3.868)
rs4553808	203866221	AA	AG	GG	Additive	AA vs.AG vs. GG	0.059	NA	
RF+	CTLA4	78	13	1	Dominant	AA vs. AG + GG	0.589	0.748	(0.260–2.152)
		76%	81%	25%	Recessive	AA + AG vs. GG	0.049*	0.103	(0.010–1.026)
RF−		25	3	3	Homozygous	AA vs. GG	0.054	0.107	(0.011–1.074)
		24%	19%	75%	Heterozygous	AA vs. AG	0.760	1.389	(0.366–5.271)
rs231775	203867991	GG	AG	AA	Additive	GG vs. AG vs. AA	0.039*	NA	
RF+	CTLA4	42	34	9	Dominant	GG vs. AG + AA	0.830	1.097	(0.472–2.550)
		74%	85%	53%	Recessive	GG + AG vs. AA	0.036*	0.311	(0.107–0.904)
RF−		15	6	8	Homozygous	GG vs. AA	0.105	0.402	(0.131–1.232)
		26%	15%	47%	Heterozygous	GG vs. AG	0.183	2.024	(0.709–5.779)
rs41386349	241851697	GG	AG	AA	Additive	GG vs. AG vs. AA	0.110	NA	
ACCP +	PDCD1	8	8	3	Dominant	GG vs. AG + AA	0.050*	2.667	(0.978–7.270)
		11%	23%	33%	Recessive	GG + AG vs. AA	0.164	2.844	(0.645–12.546)
ACCP−		64	27	6	Homozygous	GG vs. AA	0.100	4.000	(0.833–19.202)
		89%	77%	67%	Heterozygous	GG vs. AG	0.110	2.370	(0.806–6.968)
rs3181096	203705369	CC	CT	TT	Additive	CC vs. CT vs. TT	0.022*	NA	
CRP+	CD28	52	20	3	Dominant	CC vs. CT + TT	0.013*	0.391	(0.186–0.825)
		69%	44%	75%	Recessive	CC + CT vs. TT	1.000	2.000	(0.202–19.798)
CRP−		23	25	1	Homozygous	CC vs. TT	1.000	1.327	(0.131–13.445)
		31%	56%	25%	Heterozygous	CC vs. CT	0.007*	0.354	(0.165–0.761)
rs3181098	203705655	GG	AG	AA	Additive	GG vs. AG vs. AA	0.008*	NA	
CRP+	CD28	54	18	3	Dominant	GG vs. AG + AA	0.005*	0.344	(0.162–0.731)
		70%	42%	75%	Recessive	GG + AG vs. AA	1.000	2.000	(0.202–19.798)
CRP−		23	25	1	Homozygous	GG vs. AA	1.000	1.278	(0.126–12.940)
		30%	58%	25%	Heterozygous	GG vs. AG	0.002*	0.307	(0.141–0.668)
rs28688913	203705805	CC	CT	TT	Additive	CC vs. CT vs. TT	0.107	NA	
CRP+	CD28	48	24	3	Dominant	CC vs. CT + TT	0.034*	2.500	(1.054–5.927)
		55%	75%	75%	Recessive	CC + CT vs. TT	1.000	2.000	(0.202–19.798)
CRP−		40	8	1	Homozygous	CC vs. TT	0.626	2.500	(0.250–24.979)
		45%	25%	25%	Heterozygous	CC vs. CT	0.043*	2.500	(1.013–6.171)
rs733618	203866221	TT	CT	CC	Additive	TT vs.TC vs.CC	0.017*	NA	
CRP+	CTLA4	23	34	17	Dominant	TT vs. TC + CC	0.414	1.376	(0.639–2.963)
		56%	77%	47%	Recessive	TT + TC vs. CC	0.041*	0.440	(0.198–0.974)
CRP−		18	10	19	Homozygous	TT vs. CC	0.437	0.700	(0.285–1.721)
		44%	23%	53%	Heterozygous	TT vs. TC	0.038*	2.661	(1.043–6.790)
rs11571316	203866366	GG	AG	AA	Additive	GG vs. AG vs. AA	0.019*	NA	
CRP+	CTLA4	61	9	4	Dominant	GG vs. AG + AA	0.009*	0.336	(0.147–0.772)
		67%	36%	57%	Recessive	GG + AG vs. AA	1.000	0.876	(0.187–4.097)
CRP−		30	16	3	Homozygous	GG vs. AA	0.685	0.656	(0.138–3.119)
		33%	64%	43%	Heterozygous	GG vs. AG	0.005*	0.277	(0.110–0.699)
rs3087243	203874196	GG	AG	AA	Additive	GG vs. AG vs. AA	0.013*	NA	
CRP+	CTLA4	58	13	4	Dominant	GG vs. AG + AA	0.005*	0.331	(0.152–0.722)
		69%	39%	57%	Recessive	GG + AG vs. AA	1.000	0.864	(0.185–4.038)
CRP−		26	20	3	Homozygous	GG vs. AA	0.675	0.598	(0.125–2.864)
		31%	61%	43%	Heterozygous	GG vs. AG	0.003*	0.291	(0.126–0.673)
rs2227981	241851121	GG	AG	AA	Additive	GG vs. AG vs. AA	0.049*	NA	
CRP+	PDCD1	32	28	7	Dominant	GG vs. AG + AA	0.027*	2.422	(1.096–5.350)
		51%	76%	58%	Recessive	GG + AG vs. AA	1.000	0.933	(0.277–3.147)
CRP−		31	9	5	Homozygous	GG vs. AA	0.632	1.356	(0.389–4.731)
		49%	24%	42%	Heterozygous	GG vs. AG	0.014*	3.014	(1.227–7.405)
rs6705653	241851407	CC	CT	TT	Additive	CC vs. CT vs. TT	0.070	NA	
CRP+	PDCD1	34	28	8	Dominant	CC vs. CT + TT	0.030*	2.329	(1.079–5.029)
		51%	74%	62%	Recessive	CC + CT vs. TT	0.863	1.110	(0.340–3.623)
CRP−		33	10	5	Homozygous	CC vs. TT	0.476	1.553	(0.460–5.237)
		49%	26%	38%	Heterozygous	CC vs. CT	0.022*	2.718	(1.143–6.464)

RF, rheumatoid factor; ACCP, anti-cyclic citrullinated peptide antibody; CRP, C-reactive protein; additive, AA vs. Aa vs. aa; dominant: AA vs. Aa + aa; recessive, AA + Aa vs. aa; homozygous, AA vs. aa; heterozygous, AA vs. Aa, where the frequency of A-allele is major in the population, and a-allele is minor. *, <0.05; NA, not applicable.

The three SNPs associated with the presence of RA were in the CTLA4 gene. rs11571315 was associated with RF based on the recessive model (TT + TC vs. CC, OR = 0.309, 95% CI = 0.107–0.896, p = 0.035), which meant that the patients with CC genotype in rs11571315 had a lower odds of RF compared to the patients with at least one T-allele (TT and TC). rs4553808 was associated with RF based on the recessive model (AA + AG vs. GG, OR = 0.103, 95% CI = 0.010–1.026, p = 0.049). rs231775 was associated with RF based on the additive model (GG vs. AG vs. AA, p = 0.039) and recessive model (GG + AG vs. AA, OR = 0.311, 95% CI = 0.107–0.904, p = 0.036).

rs41386349 of the PDCD1 gene was associated with ACCP based on the dominant model (GG vs. AG + AA, OR = 2.667, 95% CI = 0.978–7.270, p = 0.05). Compared to the GG genotype, patients with RA with at least one A-allele in rs41386349 had 2.667 times of odds of ACCP.

There were three SNPs of the CD28 gene, three SNPs of the CTLA4 gene, and two SNPs of the PDCD1 gene associated with CRP. rs3181096 of the CD28 gene was associated with CRP based on the additive model (CC vs. CT vs. TT, p = 0.022), dominant model (CC vs. CT + TT, OR = 0.391; 95% CI = 0.186–0.825, p = 0.013), and heterozygous model (CC vs. CT, OR = 0.354; 95% CI = 0.165–0.761, p = 0.007). rs3181098 of the CD28 gene was associated with CRP based on the additive model (GG vs. AG vs. AA, p = 0.008), dominant model (GG vs. AG + AA, OR = 0.344; 95% CI = 0.162–0.731, p = 0.005), and heterozygous model (GG vs. AG, OR = 0.307; 95% CI = 0.141–0.668, p = 0.002). rs28688913 of the CD28 gene was associated with CRP based on the dominant model (CC vs. CT + TT, OR = 2.500; 95% CI = 1.054–5.927, p = 0.034), and heterozygous model (CC vs. CT, OR = 2.500, 95% CI = 1.013–6.171, p = 0.043). rs733618 of the CTLA4 gene was associated with CRP based on the additive model (TT vs. CT vs. CC, p = 0.017), recessive model (TT + CT vs. CC, OR = 0.440; 95% CI = 0.198–0.974, p = 0.041), and heterozygous model (TT vs. CT, OR = 2.661; 95% CI = 1.043–6.790, p = 0.038). rs11571316 of the CTLA4 gene was associated with CRP based on the additive model (GG vs. AG vs. AA, p = 0.019), dominant model (GG vs. AG + AA, OR = 0.336; 95% CI = 0.147–0.772, p = 0.009), and heterozygous model (GG vs. AG, OR = 0.277; 95% CI = 0.110–0.699, p = 0.005). rs3087243 of the CTLA4 gene was associated with CRP based on the additive model (GG vs. AG vs. AA, p = 0.013), dominant model (GG vs. AG + AA, OR = 0.331; 95% CI = 0.152–0.722, p = 0.005), and heterozygous model (GG vs. AG, OR = 0.291; 95% CI = 0.126–0.673, p = 0.003). rs2227981 of the PDCD1 gene was associated with CRP based on the additive model (GG vs. AG vs. AA, p = 0.049), dominant model (GG vs. AG + AA, OR = 2.422; 95% CI = 1.096–5.350, p = 0.027), and heterozygous model (GG vs. AG, OR = 3.014; 95% CI = 1.227–7.405, p = 0.014). rs6705653 of the PDCD1 gene was associated with CRP based on the dominant model (GG vs. AG + AA, OR = 2.329; 95% CI = 1.079–5.029, p = 0.030) and heterozygous model (GG vs. AG, OR = 2.718; 95% CI = 1.1143–66.464, p = 0.022).

## Discussion

SNP analysis revealed that one SNP of the TNFSF4 gene, seven SNPs of the CTLA4 gene, one SNP of the CD28 gene, and four SNPs of the PDCD1 gene were associated with the onset of RA. We also investigated the association between the SNPs and immunological parameters of patients with RA. It is well known that RF and anti-citrullinated protein antibodies (ACPA) are the characteristic autoantibodies of RA. ACCP is a subset of ACPA, which has been demonstrated that it has 65%–80% sensitivity and up to 98% specificity for RA (20). CRP plays a vital role in inflammatory response. A study showed that it had a positive correlation with the severity of RA (21). Other clinical data, such as disease activity score, disease activity score by 28 joints (DAS28), and erythrocyte sedimentation rate (ESR), are dynamic according to the disease process; thus, they would not be analyzed.

We found that rs181758110 in the promoter region of the TNFSF4 gene was associated with RA, and no SNPs of the TNFSF4 gene were associated with the characteristic autoantibodies of RA, which denotes that rs181758110 of the TNFSF4 gene may not be a major SNP that contributed to the pathogenesis of RA. There is currently no relevant research on this SNP, and its function needs to be verified.

rs3181096 in the CD28 gene promoter region was associated with RA and CRP in our results. rs3181096 had been previously associated with other ADs and cancers, such as type 1 diabetes (22) and childhood acute lymphoblastic leukemia (23) in addition to RA (24), suggesting that it may play an important role in immune response. Furthermore, there were two SNPs of the CD28 gene were associated with CRP: rs3181098 and rs28688913. Our previous study showed that rs3181098 had association with graft-versus-host disease (GVHD) of post–hematopoietic stem cell transplantation (HSCT) in acute leukemia patients (25). Minculescu et al. (26) showed that CRP level could be a valid predictor of the development of steroid-refractory disease in patients who develop severe GVHD after HSCT. Thus, rs3181098 may influence the production of CRP, thereby developing GVHD.

Results showed that rs11571315 was not only associated with the onset of RA but also is RF-positive. However, after a thorough search of the literature, it was found that no other team has yet discovered the correlation between rs11571315 and RA, suggesting that rs11571315 was the RA-associated SNP specific to the Taiwan population. However, rs11571315 was susceptible to other immune disorders, such as transfusion reactions (27) and polycystic ovary syndrome (28). We found that rs733618 was associated with RA and CRP, indicating that it may involve in the inflammatory response of RA. In addition, rs733618 was recently found to be associated with the onset of RA (29) as well as Graves’ disease (30, 31) and non–small cell lung cancer (32). Similar to rs11571315, rs4553808 was associated with the onset of RA and was RF-positive. Furthermore, rs4553808 was associated with RA (29), several ADs (33–35), cancers (36, 37), and transplant prognosis (38, 39). Similar to rs733618, rs11571316 was associated with RA and CRP in our results. The literature showed that rs11571316 may increase the susceptibility of cervical cancer by increasing the expression level of the CTLA4 (40); we indicated that rs11571316 involved in RA pathogenesis and inflammatory response may result from upregulating the CTLA4 expression. rs5742909 was a common SNP in RA, which has been previously related RA in Spain, Korea, and Egypt populations (41). rs5742909 has already been reported as functional, and the SNP mutation in the rs5742909 loci would affect the expression level of CTLA4 mRNA and protein (42, 43). rs16840252 was associated with RA in our result, but it showed no association in Chinese Han population (44). Identically, the literature (44) showed that rs16840252 was related to RF, but it had no significance in our results. This may emphasize that RA is influenced by other factor, such as the environment. Recently, Aslam et al. (45) observed a novel correlation of rs11571319 to RA in the Pakistani popuation. Before that, rs11571319 has not been reported previously related to RA risk, and it had just been reported to be susceptible to primary biliary cirrhosis (46), asthma (47), and so on.Regarding the PDCD1 gene, there is currently no literature on the association between rs5839828 and diseases, but another significant SNP of this gene, rs36084323, was associated with the risk of RA (48) and cancers (49). These two SNPs were discovered in the PDCD1 gene promoter region. If the allele in rs36084323 was G, then the promoter activity of the PDCD1 gene would be significantly higher than if the allele was A (50) and GG genotype of rs36084323 would had higher mRNA level of PDCD1 compared to AA genotype (48), which indicates that rs36084323 may involve in the development of RA through affecting the transcription level of PDCD1. rs2227982 and rs10204525 were discovered in the exon 5 region. Although there was no literature about the association between these two SNPs and RA, the GG genotype of rs2227982 and the CC genotype of rs10204525 were protective factors for hepatitis B virus infection (51), and the CT genotype of rs10204525 was associated with juvenile idiopathic arthritis (52), suggesting that these SNPs may play a role in inflammation or infection.

Moreover, haplotype analysis revealed six SNPs with high LD in the CTLA4 gene, including rs11571316, rs62182595, rs16840252, rs5742909, rs23177, and rs3087243. These six SNPs were found to be significantly associated with RA in several haplotypes. Although rs62182595, rs231775, and rs3087243 did not show a statistically significant association in individual SNP analysis, they did in haplotype analysis. This suggested that there could be an interaction between these SNPs that cause RA. Thus, the function of the RA-predisposing CTLA4 haplotype needs to be investigated further.

Following a review of the literature, it was found that these RA-associated SNPs were also related to other ADs and cancers, suggesting that these SNPs play a vital role in the immune response. In addition, we found that the association of several SNPS with RA risk was dependent on RF, ACCP, and CRP status. These immune regulatory genes are involved in the regulation of T-cell activation (6), and most of the aforementioned SNPs were found in the promoter region and 3′UTR, suggesting that these significant SNPs may affect transcription factor binding sites or interfere with mRNA stability, resulting in gene expression alterations that lead to disease (53, 54). In the future, it should be investigated whether SNP variation directly affects gene expression or protein function and leads to abnormal CD4 T-cell function to determine the role of these SNPs of immune regulatory genes in the pathogenesis of RA.

### Limitation

In this study, we did not thoroughly investigate the entire genome. Because T-cell activation is regulated by the additive effect of co-stimulatory and co-inhibitory molecules and promoter SNPs can affect gene expression, the promoter region of genes was extensively discussed in this paper. Furthermore, the hot SNPs from many kinds of literature about ADs or cancers were selected as candidate SNPs for discussion.

## Data availability statement

The datasets presented in this study can be found in online repositories. The names of the repository/repositories and accession number(s) can be found in the article/**Supplementary Material**.

## Ethics statement

The studies involving human participants were reviewed and approved by institutional review board (IRB) of Chang Gung Memorial Hospital (CGMH; IRB no. 202002097B0 and 202102018B0C601). The patients/participants provided their written informed consent to participate in this study.

## Author contributions

D-PC conceived and designed the experiments. Y-HW reviewed and wrote the final draft. W-TL and FP-H performed the experiments and analyzed and interpreted data. K-HY wrote draft of the manuscript and provided samples. All authors read and approved the final manuscript.
