# Supplementary material for: Exploration of the association between the single-nucleotide polymorphism of co-stimulatory system and rheumatoid arthritis
Source: Front Immunol. 2023 Jun 29;14:1123832. doi: 10.3389/fimmu.2023.1123832 (PMC10344454; doi:10.3389/fimmu.2023.1123832)
Supplement: Supplementary file 1 [file DataSheet_1.docx]

Supplementary Material

**Supplementary Table 1.** TNFSF4 genotypes frequencies in RA cases and healthy controls.

| **SNP** | **Gene position** | **No. of patients (%)** | | | **Model** | | **Logistic regression p** | **OR (95 % CI)** | | | | | |
| --- | --- | --- | --- | --- | --- | --- | --- | --- | --- | --- | --- | --- | --- |
| rs181758110 | 173208023 | GG | GA | AA | Additive | GG vs. GA vs. AA | 0.038* | NA |  |  |  |  |  |
| control | TNFSF4 | 96 | 4 | 0 | Dominant | GG vs. GA+AA | 0.038* | NA |  |  |  |  |  |
|  |  | 44% | 100% | 0% | Recessive | GG+GA vs. AA | NA | NA |  |  |  |  |  |
| RA |  | 124 | 0 | 0 | Homozygous | GG vs. AA | NA | NA |  |  |  |  |  |
|  |  | 56% | 0% | 0% | Heterozygous | GG vs. GA | 0.038* | NA |  |  |  |  |  |
|  |  |  |  |  |  |  |  |  |  |  |  |  |  |
| rs45454293 | 173208097 | CC | CT | TT | Additive | CC vs. CT vs. TT | 0.788 | NA |  |  |  |  |  |
| control | TNFSF4 | 71 | 26 | 3 | Dominant | CC vs. CT+TT | 0.691 | 0.888 | ( | 0.493 | - | 1.598 | ) |
|  |  | 44% | 48% | 38% | Recessive | CC+CT vs. TT | 0.734 | 1.359 | ( | 0.317 | - | 5.828 | ) |
| RA |  | 91 | 28 | 5 | Homozygous | CC vs. TT | 1.000 | 1.300 | ( | 0.301 | - | 5.625 | ) |
|  |  | 56% | 52% | 63% | Heterozygous | CC vs. CT | 0.580 | 0.840 | ( | 0.453 | - | 1.558 | ) |
|  |  |  |  |  |  |  |  |  |  |  |  |  |  |
| rs1234314 | 173208253 | CC | CG | GG | Additive | CC vs.CG vs. GG | 0.179 | NA |  |  |  |  |  |
| control | TNFSF4 | 33 | 55 | 12 | Dominant | CC vs. CG+GG | 0.259 | 0.729 | ( | 0.421 | - | 1.263 | ) |
|  |  | 40% | 51% | 36% | Recessive | CC+CG vs. GG | 0.300 | 1.495 | ( | 0.696 | - | 3.210 | ) |
| RA |  | 50 | 53 | 21 | Homozygous | CC vs. GG | 0.735 | 1.155 | ( | 0.501 | - | 2.661 | ) |
|  |  | 60% | 49% | 64% | Heterozygous | CC vs. CG | 0.125 | 0.636 | ( | 0.356 | - | 1.135 | ) |

**Supplementary Table 2.** **CD28 genotypes frequencies in RA cases and healthy controls.**

| **SNP** | **Gene position** | **No. of patients (%)** | | | **Model** | **Model** | **Logistic regression p** | **OR (95 % CI)** | | | | | |
| --- | --- | --- | --- | --- | --- | --- | --- | --- | --- | --- | --- | --- | --- |
| rs1879877 | 203705277 | TT | GT | GG | Additive | TT vs.GT vs.GG | 0.502 | NA |  |  |  |  |  |
| control | CD28 | 33 | 46 | 20 | Dominant | TT vs. GT+GG | 0.480 | 0.819 | ( | 0.471 | - | 1.425 | ) |
|  |  | 41% | 44% | 53% | Recessive | GG vs. TT+GT | 0.262 | 0.671 | ( | 0.333 | - | 1.351 | ) |
| RA |  | 47 | 59 | 18 | Homozygous | TT vs. GG | 0.245 | 0.632 | ( | 0.291 | - | 1.374 | ) |
|  |  | 59% | 56% | 47% | Heterozygous | TT vs. GT | 0.727 | 0.901 | ( | 0.500 | - | 1.623 | ) |
|  |  |  |  |  |  |  |  |  |  |  |  |  |  |
| rs3181096 | 203705369 | CC | CT | TT | Additive | CC vs. CT vs. TT | 0.101 | NA |  |  |  |  |  |
| control | CD28 | 58 | 31 | 10 | Dominant | CC vs. CT+TT | 0.774 | 0.924 | ( | 0.540 | - | 1.580 | ) |
|  |  | 44% | 41% | 71% | Recessive | CC+CT vs. TT | 0.035* | 0.297 | ( | 0.090 | - | 0.980 | ) |
| RA |  | 75 | 45 | 4 | Homozygous | CC vs. TT | 0.047* | 0.309 | ( | 0.920 | - | 1.040 | ) |
|  |  | 56% | 59% | 29% | Heterozygous | CC vs. CT | 0.692 | 1.123 | ( | 0.630 | - | 1.990 | ) |
|  |  |  |  |  |  |  |  |  |  |  |  |  |  |
| rs3181097 | 203705416 | GG | AG | AA | Additive | GG vs. AG vs. AA | 0.704 | NA |  |  |  |  |  |
| control | CD28 | 31 | 50 | 18 | Dominant | GG vs. AG+AA | 0.441 | 1.257 | ( | 0.702 | - | 2.250 | ) |
|  |  | 48% | 42% | 45% | Recessive | GG +AG vs.AA | 0.932 | 0.971 | ( | 0.488 | - | 1.931 | ) |
| RA |  | 33 | 69 | 22 | Homozygous | GG vs. AA | 0.733 | 1.148 | ( | 0.520 | - | 2.537 | ) |
|  |  | 52% | 58% | 55% | Heterozygous | GG vs. AG | 0.404 | 1.296 | ( | 0.704 | - | 2.387 | ) |
|  |  |  |  |  |  |  |  |  |  |  |  |  |  |
| rs3181098 | 203705655 | GG | AG | AA | Additive | GG vs. AG vs. AA | 0.154 | NA |  |  |  |  |  |
| control | CD28 | 61 | 29 | 9 | Dominant | GG vs. AG+AA | 0.941 | 0.980 | ( | 0.569 | - | 1.688 | ) |
|  |  | 44% | 40% | 69% | Recessive | GG+AG vs. AA | 0.063 | 0.333 | ( | 0.099 | - | 1.117 | ) |
| RA |  | 77 | 43 | 4 | Homozygous | GG vs. AA | 0.084 | 0.352 | ( | 0.103 | - | 1.198 | ) |
|  |  | 56% | 60% | 31% | Heterozygous | GG vs. AG | 0.585 | 1.175 | ( | 0.659 | - | 2.095 | ) |
|  |  |  |  |  |  |  |  |  |  |  |  |  |  |
| rs28688913 | 203705805 | CC | CT | TT | Additive | CC vs. CT vs. TT | 0.883 | NA |  |  |  |  |  |
| control | CD28 | 71 | 23 | 4 | Dominant | CC vs. CT+TT | 0.808 | 1.076 | ( | 0.597 | - | 1.938 | ) |
|  |  | 45% | 42% | 50% | Recessive | CC+CT vs. TT | 0.734 | 0.783 | ( | 0.191 | - | 3.215 | ) |
| RA |  | 88 | 32 | 4 | Homozygous | CC vs. TT | 1.000 | 0.807 | ( | 0.195 | - | 3.340 | ) |
|  |  | 55% | 58% | 50% | Heterozygous | CC vs. CT | 0.715 | 1.123 | ( | 0.604 | - | 2.087 | ) |
|  |  |  |  |  |  |  |  |  |  |  |  |  |  |
| rs56228674 | 203729436 | CC | CT | TT | Additive | CC vs. CT vs. TT | 0.705 | 1.165 | ( | 0.528 | - | 2.570 | ) |
| control | CD28 | 88 | 12 | 0 | Dominant | CC vs. CT+TT | 0.705 | 1.165 | ( | 0.528 | - | 2.570 | ) |
|  |  | 45% | 41% | 0% | Recessive | CC+CT vs. TT | NA | NA |  |  |  |  |  |
| RA |  | 107 | 17 | 0 | Homozygous | CC vs. TT | NA | NA |  |  |  |  |  |
|  |  | 55% | 59% | 0% | Heterozygous | CC vs. CT | 0.705 | 1.165 | ( | 0.528 | - | 2.570 | ) |
|  |  |  |  |  |  |  |  |  |  |  |  |  |  |
| rs3116496 | 203729789 | TT | CT | CC | Additive | TT vs. CT vs.CC | 0.700 | NA |  |  |  |  |  |
| control | CD28 | 72 | 27 | 1 | Dominant | TT vs. CT+CC | 0.923 | 0.971 | ( | 0.539 | - | 1.750 | ) |
|  |  | 44% | 47% | 25% | Recessive | TT+CT vs. CC | 0.630 | 2.455 | ( | 0.251 | - | 23.966 | ) |
| RA |  | 90 | 31 | 3 | Homozygous | TT vs. CC | 0.631 | 2.400 | ( | 0.244 | - | 23.565 | ) |
|  |  | 56% | 53% | 75% | Heterozygous | TT vs. CT | 0.782 | 0.919 | ( | 0.503 | - | 1.677 | ) |
| *: P<0.05; NA: not applicable | | | | | | | | | | | | | |

**Supplementary Table 3. CTLA4 genotypes frequencies in RA cases and healthy controls.**

| **SNP** | **Gene position** | **No. of patients (%)** | | | **Model** | | **Logistic regression p** | **OR (95 % CI)** | | | | | |
| --- | --- | --- | --- | --- | --- | --- | --- | --- | --- | --- | --- | --- | --- |
| rs11571315 | 203866178 | TT | CT | CC | Additive | TT vs.TC vs.CC | 0.117 | NA |  |  |  |  |  |
| control | CTLA4 | 47 | 41 | 12 | Dominant | TT vs. TC+CC | 0.105 | 0.643 | ( | 0.376 | - | 1.098 | ) |
|  |  | 41% | 55% | 41% | Recessive | TT+TC vs. CC | 0.619 | 1.222 | ( | 0.554 | - | 2.699 | ) |
| RA |  | 69 | 33 | 17 | Homozygous | TT vs. CC | 0.933 | 0.965 | ( | 0.422 | - | 2.206 | ) |
|  |  | 59% | 45% | 59% | Heterozygous | TT vs. TC | 0.045* | 0.548 | ( | 0.304 | - | 0.989 | ) |
|  |  |  |  |  |  |  |  |  |  |  |  |  |  |
| rs733618 | 203866221 | TT | CT | CC | Additive | TT vs.TC vs.CC | 0.110 | NA |  |  |  |  |  |
| control | CTLA4 | 36 | 46 | 18 | Dominant | TT vs. TC+CC | 0.742 | 1.098 | ( | 0.630 | - | 1.910 | ) |
|  |  | 47% | 51% | 33% | Recessive | TT+TC vs. CC | 0.043* | 1.929 | ( | 1.020 | - | 3.670 | ) |
| RA |  | 41 | 44 | 36 | Homozygous | TT vs. CC | 0.125 | 1.756 | ( | 0.850 | - | 3.610 | ) |
|  |  | 53% | 49% | 67% | Heterozygous | TT vs. TC | 0.574 | 0.840 | ( | 0.460 | - | 1.550 | ) |
|  |  |  |  |  |  |  |  |  |  |  |  |  |  |
| rs4553808 | 203866221 | AA | AG | GG | Additive | AA vs.AG vs. GG | 0.035* | NA |  |  |  |  |  |
| control | CTLA4 | 77 | 23 | 0 | Dominant | AA vs. AG+GG | 0.205 | 0.650 | ( | 0.330 | - | 1.270 | ) |
|  |  | 43% | 59% | 0% | Recessive | AA+AG vs. GG | 0.130 | NA |  |  |  |  |  |
| RA |  | 103 | 16 | 4 | Homozygous | AA vs. GG | 0.141 | NA |  |  |  |  |  |
|  |  | 57% | 41% | 100% | Heterozygous | AA vs. AG | 0.066 | 0.520 | ( | 0.260 | - | 1.050 | ) |
|  |  |  |  |  |  |  |  |  |  |  |  |  |  |
| rs11571316 | 203866366 | GG | AG | AA | Additive | GG vs. AG vs. AA | 0.048* | NA |  |  |  |  |  |
| control | CTLA4 | 60 | 35 | 5 | Dominant | GG vs. AG+AA | 0.026* | 0.527 | ( | 0.300 | - | 0.930 | ) |
|  |  | 40% | 58% | 42% | Recessive | GG+AG vs. AA | 0.820 | 1.147 | ( | 0.350 | - | 3.730 | ) |
| RA |  | 91 | 25 | 7 | Homozygous | GG vs. AA | 1.000 | 0.923 | ( | 0.280 | - | 3.040 | ) |
|  |  | 60% | 42% | 58% | Heterozygous | GG vs. AG | 0.014* | 0.471 | ( | 0.260 | - | 0.870 | ) |
|  |  |  |  |  |  |  |  |  |  |  |  |  |  |
| rs62182595 | 203866465 | GG | AG | AA | Additive | GG vs. AG vs. AA | 0.220 | NA |  |  |  |  |  |
| control | CTLA4 | 78 | 21 | 1 | Dominant | GG vs. AG+AA | 0.369 | 0.737 | ( | 0.378 | - | 1.436 | ) |
|  |  | 44% | 55% | 20% | Recessive | GG+AG vs. AA | 0.382 | 3.356 | ( | 0.369 | - | 30.516 | ) |
| RA |  | 101 | 17 | 4 | Homozygous | GG vs. AA | 0.393 | 3.089 | ( | 0.338 | - | 28.191 | ) |
|  |  | 56% | 45% | 80% | Heterozygous | GG vs. AG | 0.189 | 0.625 | ( | 0.309 | - | 1.265 | ) |
|  |  |  |  |  |  |  |  |  |  |  |  |  |  |
| rs16840252 | 203866796 | CC | CT | TT | Additive | CC vs. CT vs. TT | 0.007* | NA |  |  |  |  |  |
| control | CTLA4 | 75 | 25 | 0 | Dominant | CC vs. CT+TT | 0.051 | 0.514 | ( | 0.260 | - | 1.010 | ) |
|  |  | 42% | 64% | 0% | Recessive | CC+CT vs. TT | 0.130 | NA |  |  |  |  |  |
| RA |  | 105 | 14 | 4 | Homozygous | CC vs. TT | 0.147 | NA |  |  |  |  |  |
|  |  | 58% | 36% | 100% | Heterozygous | CC vs. CT | 0.011* | 0.400 | ( | 0.200 | - | 0.820 | ) |
|  |  |  |  |  |  |  |  |  |  |  |  |  |  |
| rs5742909 | 203867624 | CC | CT | TT | Additive | CC vs. CT vs. TT | 0.040* | NA |  |  |  |  |  |
| control | CTLA4 | 71 | 29 | 0 | Dominant | CC vs. CT+TT | 0.340 | 0.747 | ( | 0.410 | - | 1.360 | ) |
|  |  | 43% | 55% | 0% | Recessive | CC+CT vs. TT | 0.067 | NA |  |  |  |  |  |
| RA |  | 95 | 24 | 5 | Homozygous | CC vs. TT | 0.077 | NA |  |  |  |  |  |
|  |  | 57% | 45% | 100% | Heterozygous | CC vs. CT | 0.128 | 0.619 | ( | 0.330 | - | 1.150 | ) |
|  |  |  |  |  |  |  |  |  |  |  |  |  |  |
| rs231775 | 203867991 | GG | AG | AA | Additive | GG vs. AG vs. AA | 0.190 | NA |  |  |  |  |  |
| control | CTLA4 | 42 | 46 | 10 | Dominant | GG vs. AG+AA | 0.299 | 0.750 | ( | 0.436 | - | 1.291 | ) |
|  |  | 42% | 53% | 37% | Recessive | GG+AG vs. AA | 0.305 | 1.542 | ( | 0.671 | - | 3.546 | ) |
| RA |  | 57 | 40 | 17 | Homozygous | GG vs. AA | 0.614 | 1.253 | ( | 0.521 | - | 3.011 | ) |
|  |  | 58% | 47% | 63% | Heterozygous | GG vs. AG | 0.133 | 0.641 | ( | 0.358 | - | 1.146 | ) |
|  |  |  |  |  |  |  |  |  |  |  |  |  |  |
| rs3087243 | 203874196 | GG | AG | AA | Additive | GG vs. AG vs. AA | 0.486 | NA |  |  |  |  |  |
| control | CTLA4 | 61 | 34 | 5 | Dominant | GG vs. AG+AA | 0.294 | 0.745 | ( | 0.429 | - | 1.292 | ) |
|  |  | 42% | 51% | 42% | Recessive | GG+AG vs. AA | 0.831 | 1.137 | ( | 0.350 | - | 3.696 | ) |
| RA |  | 84 | 33 | 7 | Homozygous | GG vs. AA | 0.978 | 1.017 | ( | 0.308 | - | 3.355 | ) |
|  |  | 58% | 49% | 58% | Heterozygous | GG vs. AG | 0.238 | 0.705 | ( | 0.394 | - | 1.261 | ) |
|  |  |  |  |  |  |  |  |  |  |  |  |  |  |
| rs11571319 | 203874215 | GG | AG | AA | Additive | GG vs. GA vs. AA | <0.001* | NA |  |  |  |  |  |
| control | CTLA4 | 61 | 38 | 1 | Dominant | GG vs. GA+AA | 0.048* | 0.567 | ( | 0.320 | - | 1.000 | ) |
|  |  | 40% | 68% | 6% | Recessive | GG+GA vs. AA | 0.001* | 13.624 | ( | 1.770 | - | 105.030 | ) |
| RA |  | 91 | 18 | 15 | Homozygous | GG vs. AA | 0.008* | 10.060 | ( | 1.290 | - | 78.110 | ) |
|  |  | 60% | 32% | 94% | Heterozygous | GG vs. GA | <0.001* | 0.318 | ( | 0.170 | - | 0.610 | ) |
| *: P<0.05; NA: not applicable | | | | | | | | | | | | | |

**Supplementary Table 4.** PDCD1 genotypes frequencies in RA cases and healthy controls.

| **SNP** | **Gene position** | **No. of patients (%)** | | | **Model** | | **Logistic regression p** | **OR (95 % CI)** | | | | | |
| --- | --- | --- | --- | --- | --- | --- | --- | --- | --- | --- | --- | --- | --- |
| rs10204525 | 241850169 | TT | CT | CC | Additive | TT vs. CT vs.CC | 0.069 | NA |  |  |  |  |  |
| control | PDCD1 | 65 | 31 | 4 | Dominant | TT vs. CT+CC | 0.024* | 1.857 | ( | 1.080 | - | 3.190 | ) |
|  |  | 51% | 35% | 44% | Recessive | TT+CT vs. CC | 1.000 | 1.008 | ( | 0.264 | - | 3.859 | ) |
| RA |  | 62 | 57 | 5 | Homozygous | TT vs. CC | 0.743 | 1.310 | ( | 0.340 | - | 5.110 | ) |
|  |  | 49% | 65% | 56% | Heterozygous | TT vs. CT | 0.021* | 1.928 | ( | 1.100 | - | 3.370 | ) |
|  |  |  |  |  |  |  |  |  |  |  |  |  |  |
| rs56029561 | 241850737 | DEL | DEL/CAG | CAG | Additive | DEL vs. DEL/CAG vs.CAG | 0.535 | 1.366 | ( | 0.509 | - | 3.667 | ) |
| control | PDCD1 | 93 | 7 | 0 | Dominant | DEL vs.DEL/CAG+CAG | 0.535 | 1.366 | ( | 0.509 | - | 3.667 | ) |
|  |  | 47% | 39% | 0% | Recessive | DEL+DEL/CAG vs.CAG | NA | NA |  |  |  |  |  |
| RA |  | 107 | 11 | 0 | Homozygous | DEL vs.CAG | NA | NA |  |  |  |  |  |
|  |  | 54% | 61% | 0% | Heterozygous | DEL vs.DEL/CAG | 0.535 | 1.366 | ( | 0.509 | - | 3.667 | ) |
|  |  |  |  |  |  |  |  |  |  |  |  |  |  |
| rs2227981 | 241851121 | GG | AG | AA | Additive | GG vs. AG vs. AA | 0.404 | NA |  |  |  |  |  |
| control | PDCD1 | 64 | 28 | 7 | Dominant | GG vs. AG+AA | 0.192 | 1.441 | ( | 0.832 | - | 2.496 | ) |
|  |  | 49% | 41% | 37% | Recessive | GG+AG vs. AA | 0.421 | 1.488 | ( | 0.562 | - | 3.937 | ) |
| RA |  | 66 | 40 | 12 | Homozygous | GG vs. AA | 0.313 | 1.662 | ( | 0.616 | - | 4.490 | ) |
|  |  | 51% | 59% | 63% | Heterozygous | GG vs. AG | 0.281 | 1.385 | ( | 0.766 | - | 2.506 | ) |
|  |  |  |  |  |  |  |  |  |  |  |  |  |  |
| rs2227982 | 241851281 | AA | AG | GG | Additive | AA vs.AG vs. GG | 0.139 | NA |  |  |  |  |  |
| control | PDCD1 | 38 | 46 | 15 | Dominant | AA vs. AG+GG | 0.138 | 1.531 | ( | 0.870 | - | 2.690 | ) |
|  |  | 52% | 45% | 33% | Recessive | AA+AG vs. GG | 0.078 | 1.846 | ( | 0.930 | - | 3.670 | ) |
| RA |  | 35 | 56 | 30 | Homozygous | AA vs. GG | 0.047* | 2.171 | ( | 1.000 | - | 4.700 | ) |
|  |  | 48% | 55% | 67% | Heterozygous | AA vs. AG | 0.364 | 1.322 | ( | 0.720 | - | 2.410 | ) |
|  |  |  |  |  |  |  |  |  |  |  |  |  |  |
| rs6705653 | 241851407 | CC | CT | TT | Additive | CC vs. CT vs. TT | 0.345 | NA |  |  |  |  |  |
| control | PDCD1 | 64 | 29 | 6 | Dominant | CC vs. CT+TT | 0.214 | 1.411 | ( | 0.819 | - | 2.430 | ) |
|  |  | 48% | 41% | 32% | Recessive | CC+CT vs. TT | 0.240 | 1.815 | ( | 0.664 | - | 4.963 | ) |
| RA |  | 70 | 41 | 13 | Homozygous | CC vs. TT | 0.185 | 1.981 | ( | 0.711 | - | 5.521 | ) |
|  |  | 52% | 59% | 68% | Heterozygous | CC vs. CT | 0.389 | 1.293 | ( | 0.721 | - | 2.318 | ) |
|  |  |  |  |  |  |  |  |  |  |  |  |  |  |
| rs41386349 | 241851697 | GG | AG | AA | Additive | GG vs. AG vs. AA | 0.590 | NA |  |  |  |  |  |
| control | PDCD1 | 69 | 26 | 5 | Dominant | GG vs. AG+AA | 0.340 | 1.313 | ( | 0.751 | - | 2.295 | ) |
|  |  | 47% | 41% | 36% | Recessive | GG+AG vs. AA | 0.488 | 1.487 | ( | 0.482 | - | 4.587 | ) |
| RA |  | 78 | 37 | 9 | Homozygous | GG vs. AA | 0.421 | 1.592 | ( | 0.509 | - | 4.980 | ) |
|  |  | 53% | 59% | 64% | Heterozygous | GG vs. AG | 0.449 | 1.259 | ( | 0.693 | - | 2.287 | ) |
|  |  |  |  |  |  |  |  |  |  |  |  |  |  |
| rs36084323 | 241859444 | TT | CT | CC | Additive | TT vs. CT vs.CC | 0.022* | NA |  |  |  |  |  |
| control |  | 40 | 43 | 13 | Dominant | TT vs. CT+CC | 0.013* | 2.054 | ( | 1.160 | - | 3.640 | ) |
|  |  | 56% | 41% | 30% | Recessive | TT+CT vs. CC | 0.048* | 2.038 | ( | 1.000 | - | 4.160 | ) |
| RA |  | 32 | 62 | 30 | Homozygous | TT vs. CC | 0.008* | 2.885 | ( | 1.300 | - | 6.420 | ) |
|  |  | 44% | 59% | 70% | Heterozygous | TT vs. CT | 0.056 | 1.802 | ( | 0.980 | - | 3.300 | ) |
|  |  |  |  |  |  |  |  |  |  |  |  |  |  |
| rs5839828 | 241859601 | DEL | DEL/G | GG | Additive | DEL vs. DEL/G vs.GG | 0.014* | NA |  |  |  |  |  |
| control |  | 50 | 41 | 5 | Dominant | DEL vs. DEL/G+GG | 0.014* | 1.976 | ( | 1.150 | - | 3.400 | ) |
|  |  | 53% | 40% | 22% | Recessive | DEL+DEL/G vs.GG | 0.025* | 3.091 | ( | 1.100 | - | 8.650 | ) |
| RA |  | 44 | 62 | 18 | Homozygous | DEL vs.GG | 0.007* | 4.091 | ( | 1.400 | - | 11.930 | ) |
|  |  | 47% | 60% | 78% | Heterozygous | DEL vs. DEL/G | 0.060 | 1.718 | ( | 0.980 | - | 3.030 | ) |
| *: P<0.05; NA: not applicable | | | | | | | | | | | | | |

**Supplementary Table 5. The association between SNPs and RF.**

| **SNP** | **Gene position** | **No. of patients (%)** | | | **Model** | | **Logistic regression p** | **OR (95 % CI)** | | | | | |
| --- | --- | --- | --- | --- | --- | --- | --- | --- | --- | --- | --- | --- | --- |
| rs181758110 | 173208023 | GG | GA | AA | Additive | GG vs. GA vs. AA | NA | NA |  |  |  |  |  |
| RF+ | TNFSF4 | 92 | 0 | 0 | Dominant | GG vs. GA+AA | NA | NA |  |  |  |  |  |
|  |  | 74% | 0% | 0% | Recessive | GG+GA vs. AA | NA | NA |  |  |  |  |  |
| RF- |  | 32 | 0 | 0 | Homozygous | GG vs. AA | NA | NA |  |  |  |  |  |
|  |  | 26% | 0% | 0% | Heterozygous | GG vs. GA | NA | NA |  |  |  |  |  |
|  |  |  |  |  |  |  |  |  |  |  |  |  |  |
| rs45454293 | 173208097 | CC | CT | TT | Additive | CC vs. CT vs. TT | 0.265 | NA |  |  |  |  |  |
| RF+ | TNFSF4 | 71 | 18 | 3 | Dominant | CC vs. CT+TT | 0.106 | 0.493 | ( | 0.207 | - | 1.171 | ) |
|  |  | 78% | 64% | 60% | Recessive | CC+CT vs. TT | 0.603 | 0.506 | ( | 0.081 | - | 3.172 | ) |
| RF- |  | 20 | 10 | 2 | Homozygous | CC vs. TT | 0.322 | 0.423 | ( | 0.066 | - | 2.705 | ) |
|  |  | 22% | 36% | 40% | Heterozygous | CC vs. CT | 0.143 | 0.507 | ( | 0.202 | - | 1.270 | ) |
|  |  |  |  |  |  |  |  |  |  |  |  |  |  |
| rs1234314 | 173208253 | CC | CG | GG | Additive | CC vs.CG vs. GG | 0.433 | NA |  |  |  |  |  |
| RF+ | TNFSF4 | 40 | 38 | 14 | Dominant | CC vs. CG+GG | 0.225 | 0.591 | ( | 0.252 | - | 1.388 | ) |
|  |  | 80% | 72% | 67% | Recessive | CC+CG vs. GG | 0.387 | 0.641 | ( | 0.233 | - | 1.765 | ) |
| RF- |  | 10 | 15 | 7 | Homozygous | CC vs. GG | 0.230 | 0.500 | ( | 0.160 | - | 1.566 | ) |
|  |  | 20% | 28% | 33% | Heterozygous | CC vs. CG | 0.326 | 0.633 | ( | 0.254 | - | 1.581 | ) |
|  |  |  |  |  |  |  |  |  |  |  |  |  |  |
| rs1879877 | 203705277 | TT | GT | GG | Additive | TT vs.GT vs.GG | 0.664 | NA |  |  |  |  |  |
| RF+ | CD28 | 37 | 42 | 13 | Dominant | TT vs. GT+GG | 0.368 | 0.676 | ( | 0.287 | - | 1.590 | ) |
|  |  | 79% | 71% | 72% | Recessive | GG vs. TT+GT | 0.779 | 0.889 | ( | 0.290 | - | 2.724 | ) |
| RF- |  | 10 | 17 | 5 | Homozygous | TT vs. GG | 0.743 | 0.703 | ( | 0.202 | - | 2.442 | ) |
|  |  | 21% | 29% | 28% | Heterozygous | TT vs. GT | 0.376 | 0.668 | ( | 0.272 | - | 1.638 | ) |
|  |  |  |  |  |  |  |  |  |  |  |  |  |  |
| rs3181096 | 203705369 | CC | CT | TT | Additive | CC vs. CT vs. TT | 0.782 | NA |  |  |  |  |  |
| RF+ | CD28 | 54 | 35 | 3 | Dominant | CC vs. CT+TT | 0.490 | 1.343 | ( | 0.580 | - | 3.110 | ) |
|  |  | 72% | 78% | 75% | Recessive | CC+CT vs. TT | 1.000 | 1.045 | ( | 0.105 | - | 10.420 | ) |
| RF- |  | 21 | 10 | 1 | Homozygous | CC vs. TT | 1.000 | 1.167 | ( | 0.115 | - | 11.855 | ) |
|  |  | 28% | 22% | 25% | Heterozygous | CC vs. CT | 0.484 | 1.361 | ( | 0.573 | - | 3.232 | ) |
|  |  |  |  |  |  |  |  |  |  |  |  |  |  |
| rs3181097 | 203705416 | GG | AG | AA | Additive | GG vs. AG vs. AA | 0.183 | NA |  |  |  |  |  |
| RF+ | CD28 | 26 | 47 | 19 | Dominant | GG vs. AG+AA | 0.481 | 0.711 | ( | 0.274 | - | 1.844 | ) |
|  |  | 79% | 68% | 86% | Recessive | GG +AG vs.AA | 0.150 | 2.516 | ( | 0.692 | - | 9.153 | ) |
| RF- |  | 7 | 22 | 3 | Homozygous | GG vs. AA | 0.723 | 1.705 | ( | 0.390 | - | 7.462 | ) |
|  |  | 21% | 32% | 14% | Heterozygous | GG vs. AG | 0.264 | 0.575 | ( | 0.217 | - | 1.527 | ) |
|  |  |  |  |  |  |  |  |  |  |  |  |  |  |
| rs3181098 | 203705655 | GG | AG | AA | Additive | GG vs. AG vs. AA | 0.890 | NA |  |  |  |  |  |
| RF+ | CD28 | 56 | 33 | 3 | Dominant | GG vs. AG+AA | 0.633 | 1.227 | ( | 0.529 | - | 2.846 | ) |
|  |  | 73% | 77% | 75% | Recessive | GG+AG vs. AA | 1.000 | 1.045 | ( | 0.105 | - | 10.420 | ) |
| RF- |  | 21 | 10 | 1 | Homozygous | GG vs. AA | 1.000 | 1.125 | ( | 0.111 | - | 11.426 | ) |
|  |  | 27% | 23% | 25% | Heterozygous | GG vs. AG | 0.630 | 1.238 | ( | 0.520 | - | 2.946 | ) |
|  |  |  |  |  |  |  |  |  |  |  |  |  |  |
| rs28688913 | 203705805 | CC | CT | TT | Additive | CC vs. CT vs. TT | 0.248 | NA |  |  |  |  |  |
| RF+ | CD28 | 67 | 21 | 4 | Dominant | CC vs. CT+TT | 0.440 | 0.712 | ( | 0.301 | - | 1.687 | ) |
|  |  | 76% | 66% | 100% | Recessive | CC+CT vs. TT | 0.572 | NA |  |  |  |  | ) |
| RF- |  | 21 | 11 | 0 | Homozygous | CC vs. TT | 0.570 | NA |  |  |  |  | ) |
|  |  | 24% | 34% | 0% | Heterozygous | CC vs. CT | 0.250 | 0.598 | ( | 0.248 | - | 1.441 | ) |
|  |  |  |  |  |  |  |  |  |  |  |  |  |  |
| rs56228674 | 203729436 | CC | CT | TT | Additive | CC vs. CT vs. TT | 0.234 | 2.922 | ( | 0.630 | - | 13.556 | ) |
| RF+ | CD28 | 77 | 15 | 0 | Dominant | CC vs. CT+TT | 0.234 | 2.922 | ( | 0.630 | - | 13.556 | ) |
|  |  | 72% | 88% | 0% | Recessive | CC+CT vs. TT | NA | NA |  |  |  |  |  |
| RF- |  | 30 | 2 | 0 | Homozygous | CC vs. TT | NA | NA |  |  |  |  |  |
|  |  | 28% | 12% | 0% | Heterozygous | CC vs. CT | 0.234 | 2.922 | ( | 0.630 | - | 13.556 | ) |
|  |  |  |  |  |  |  |  |  |  |  |  |  |  |
| rs3116496 | 203729789 | TT | CT | CC | Additive | TT vs. CT vs.CC | 0.583 | NA |  |  |  |  |  |
| RF+ | CD28 | 66 | 23 | 3 | Dominant | TT vs. CT+CC | 0.722 | 1.182 | ( | 0.471 | - | 2.965 | ) |
|  |  | 73% | 74% | 100% | Recessive | TT+CT vs. CC | 0.568 | NA |  |  |  |  | ) |
| RF- |  | 24 | 8 | 0 | Homozygous | TT vs. CC | 0.566 | NA |  |  |  |  | ) |
|  |  | 27% | 26% | 0% | Heterozygous | TT vs. CT | 0.925 | 1.045 | ( | 0.412 | - | 2.651 | ) |
|  |  |  |  |  |  |  |  |  |  |  |  |  |  |
| rs11571315 | 203866178 | TT | CT | CC | Additive | TT vs.TC vs.CC | 0.070 | NA |  |  |  |  |  |
| RF+ | CTLA4 | 53 | 27 | 9 | Dominant | TT vs. TC+CC | 0.551 | 0.776 | ( | 0.338 | - | 1.785 | ) |
|  |  | 77% | 82% | 53% | Recessive | TT+TC vs. CC | 0.035* | 0.309 | ( | 0.107 | - | 0.896 | ) |
| RF- |  | 16 | 6 | 8 | Homozygous | TT vs. CC | 0.070 | 0.340 | ( | 0.113 | - | 1.025 | ) |
|  |  | 23% | 18% | 47% | Heterozygous | TT vs. TC | 0.565 | 1.358 | ( | 0.477 | - | 3.868 | ) |
|  |  |  |  |  |  |  |  |  |  |  |  |  |  |
| rs733618 | 203866221 | TT | CT | CC | Additive | TT vs.TC vs.CC | 0.976 | NA |  |  |  |  |  |
| RF+ | CTLA4 | 30 | 33 | 27 | Dominant | TT vs. TC+CC | 0.827 | 1.100 | ( | 0.467 | - | 2.590 | ) |
|  |  | 73% | 75% | 75% | Recessive | TT+TC vs. CC | 0.919 | 1.048 | ( | 0.427 | - | 2.569 | ) |
| RF- |  | 11 | 11 | 9 | Homozygous | TT vs. CC | 0.855 | 1.100 | ( | 0.395 | - | 3.059 | ) |
|  |  | 27% | 25% | 25% | Heterozygous | TT vs. TC | 0.847 | 1.100 | ( | 0.417 | - | 2.905 | ) |
|  |  |  |  |  |  |  |  |  |  |  |  |  |  |
| rs4553808 | 203866221 | AA | AG | GG | Additive | AA vs.AG vs. GG | 0.059 | NA |  |  |  |  |  |
| RF+ | CTLA4 | 78 | 13 | 1 | Dominant | AA vs. AG+GG | 0.589 | 0.748 | ( | 0.260 | - | 2.152 | ) |
|  |  | 76% | 81% | 25% | Recessive | AA+AG vs. GG | 0.049* | 0.103 | ( | 0.010 | - | 1.026 |  |
| RF- |  | 25 | 3 | 3 | Homozygous | AA vs. GG | 0.054 | 0.107 | ( | 0.011 | - | 1.074 |  |
|  |  | 24% | 19% | 75% | Heterozygous | AA vs. AG | 0.760 | 1.389 | ( | 0.366 | - | 5.271 | ) |
|  |  |  |  |  |  |  |  |  |  |  |  |  |  |
| rs11571316 | 203866366 | GG | AG | AA | Additive | GG vs. AG vs. AA | 0.574 | NA |  |  |  |  |  |
| RF+ | CTLA4 | 68 | 19 | 4 | Dominant | GG vs. AG+AA | 0.752 | 0.864 | ( | 0.350 | - | 2.135 | ) |
|  |  | 75% | 76% | 57% | Recessive | GG+AG vs. AA | 0.375 | 0.444 | ( | 0.094 | - | 2.104 | ) |
| RF- |  | 23 | 6 | 3 | Homozygous | GG vs. AA | 0.378 | 0.451( | ( | 0.094 | - | 2.167 | ) |
|  |  | 25% | 24% | 43% | Heterozygous | GG vs. AG | 0.896 | 1.071 | ( | 0.381 | - | 3.007 | ) |
|  |  |  |  |  |  |  |  |  |  |  |  |  |  |
| rs62182595 | 203866465 | GG | AG | AA | Additive | GG vs. AG vs. AA | 0.062 | NA |  |  |  |  |  |
| RF+ | CTLA4 | 75 | 14 | 1 | Dominant | GG vs. AG+AA | 0.789 | 0.867 | ( | 0.304 | - | 2.468 | ) |
|  |  | 74% | 82% | 25% | Recessive | GG+AG vs. AA | 0.055 | 0.109 | ( | 0.011 | - | 1.085 | ) |
| RF- |  | 26 | 3 | 3 | Homozygous | GG vs. AA | 0.063 | 0.116 | ( | 0.012 | - | 1.160 | ) |
|  |  | 26% | 18% | 75% | Heterozygous | GG vs. AG | 0.559 | 1.618 | ( | 0.430 | - | 6.082 | ) |
|  |  |  |  |  |  |  |  |  |  |  |  |  |  |
| rs16840252 | 203866796 | CC | CT | TT | Additive | CC vs. CT vs. TT | 0.073 | NA |  |  |  |  |  |
| RF+ | CTLA4 | 79 | 11 | 1 | Dominant | CC vs. CT+TT | 0.561 | 0.658 | ( | 0.225 | - | 1.930 | ) |
|  |  | 75% | 79% | 25% | Recessive | CC+CT vs. TT | 0.054 | 0.107 | ( | 0.011 | - | 1.073 |  |
| RF- |  | 26 | 3 | 3 | Homozygous | CC vs. TT | 0.057 | 0.110 | ( | 0.011 | - | 1.101 |  |
|  |  | 25% | 21% | 75% | Heterozygous | CC vs. CT | 1.000 | 1.207 | ( | 0.312 | - | 4.661 | ) |
|  |  |  |  |  |  |  |  |  |  |  |  |  |  |
| rs5742909 | 203867624 | CC | CT | TT | Additive | CC vs. CT vs. TT | 0.185 | NA |  |  |  |  |  |
| RF+ | CTLA4 | 71 | 19 | 2 | Dominant | CC vs. CT+TT | 0.802 | 0.887 | ( | 0.348 | - | 2.264 | ) |
|  |  | 75% | 79% | 40% | Recessive | CC+CT vs. TT | 0.108 | 0.215 | ( | 0.034 | - | 1.349 |  |
| RF- |  | 24 | 5 | 3 | Homozygous | CC vs. TT | 0.120 | 0.225 | ( | 0.036 | - | 1.430 |  |
|  |  | 25% | 21% | 60% | Heterozygous | CC vs. CT | 0.652 | 1.285 | ( | 0.433 | - | 3.814 | ) |
|  |  |  |  |  |  |  |  |  |  |  |  |  |  |
| rs231775 | 203867991 | GG | AG | AA | Additive | GG vs. AG vs. AA | 0.039* | NA |  |  |  |  |  |
| RF+ | CTLA4 | 42 | 34 | 9 | Dominant | GG vs. AG+AA | 0.830 | 1.097 | ( | 0.472 | - | 2.550 | ) |
|  |  | 74% | 85% | 53% | Recessive | GG+AG vs. AA | 0.036* | 0.311 | ( | 0.107 | - | 0.904 | ) |
| RF- |  | 15 | 6 | 8 | Homozygous | GG vs. AA | 0.105 | 0.402 | ( | 0.131 | - | 1.232 | ) |
|  |  | 26% | 15% | 47% | Heterozygous | GG vs. AG | 0.183 | 2.024 | ( | 0.709 | - | 5.779 | ) |
|  |  |  |  |  |  |  |  |  |  |  |  |  |  |
| rs3087243 | 203874196 | GG | AG | AA | Additive | GG vs. AG vs. AA | 0.337 | NA |  |  |  |  |  |
| RF+ | CTLA4 | 61 | 27 | 4 | Dominant | GG vs. AG+AA | 0.663 | 1.299 | ( | 0.537 | - | 3.142 | ) |
|  |  | 73% | 82% | 57% | Recessive | GG+AG vs. AA | 0.373 | 0.439 | ( | 0.093 | - | 2.080 | ) |
| RF- |  | 23 | 6 | 3 | Homozygous | GG vs. AA | 0.403 | 0.503 | ( | 0.104 | - | 2.421 | ) |
|  |  | 27% | 18% | 43% | Heterozygous | GG vs. AG | 0.300 | 1.697 | ( | 0.620 | - | 4.641 | ) |
|  |  |  |  |  |  |  |  |  |  |  |  |  |  |
| rs11571319 | 203874215 | GG | AG | AA | Additive | GG vs. GA vs. AA | 0.163 | NA |  |  |  |  |  |
| RF+ | CTLA4 | 67 | 16 | 9 | Dominant | GG vs. GA+AA | 0.811 | 1.119 | ( | 0.445 | - | 2.816 | ) |
|  |  | 74% | 89% | 60% | Recessive | GG+GA vs. AA | 0.211 | 0.470 | ( | 0.153 | - | 1.444 | ) |
| RF- |  | 24 | 2 | 6 | Homozygous | GG vs. AA | 0.354 | 0.537 | ( | 0.173 | - | 1.669 | ) |
|  |  | 26% | 11% | 40% | Heterozygous | GG vs. GA | 0.231 | 2.866 | ( | 0.613 | - | 13.396 | ) |
|  |  |  |  |  |  |  |  |  |  |  |  |  |  |
| rs10204525 | 241850169 | TT | CT | CC | Additive | TT vs. CT vs.CC | 0.850 | NA |  |  |  |  |  |
| RF+ | PDCD1 | 47 | 41 | 4 | Dominant | TT vs. CT+CC | 0.681 | 0.845 | ( | 0.377 | - | 1.891 | ) |
|  |  | 76% | 72% | 80% | Recessive | TT+CT vs. CC | 1.000 | 1.409 | ( | 0.152 | - | 13.094 | ) |
| RF- |  | 15 | 16 | 1 | Homozygous | TT vs. CC | 1.000 | 1.277 | ( | 0.132 | - | 12.321 | ) |
|  |  | 24% | 28% | 20% | Heterozygous | TT vs. CT | 0.630 | 0.818 | ( | 0.360 | - | 1.856 | ) |
|  |  |  |  |  |  |  |  |  |  |  |  |  |  |
| rs56029561 | 241850737 | DEL | DEL/CAG | CAG | Additive | DEL vs. DEL/CAG vs.CAG | 0.725 | 1.673 | ( | 0.341 | - | 8.207 | ) |
| RF+ | PDCD1 | 78 | 9 | 0 | Dominant | DEL vs.DEL/CAG+CAG | 0.725 | 1.673 | ( | 0.341 | - | 8.207 | ) |
|  |  | 73% | 82% | 0% | Recessive | DEL+DEL/CAG vs.CAG | NA | NA |  |  |  |  |  |
| RF- |  | 29 | 2 | 0 | Homozygous | DEL vs.CAG | NA | NA |  |  |  |  |  |
|  |  | 27% | 18% | 0% | Heterozygous | DEL vs.DEL/CAG | 0.725 | 1.673 | ( | 0.341 | - | 8.207 | ) |
|  |  |  |  |  |  |  |  |  |  |  |  |  |  |
| rs2227981 | 241851121 | GG | AG | AA | Additive | GG vs. AG vs. AA | 0.744 | NA |  |  |  |  |  |
| RF+ | PDCD1 | 45 | 29 | 9 | Dominant | GG vs. AG+AA | 0.463 | 1.382 | ( | 0.582 | - | 3.283 | ) |
|  |  | 71% | 78% | 75% | Recessive | GG+AG vs. AA | 1.000 | 1.054 | ( | 0.265 | - | 4.194 | ) |
| RF- |  | 18 | 8 | 3 | Homozygous | GG vs. AA | 1.000 | 1.200 | ( | 0.291 | - | 4.947 | ) |
|  |  | 29% | 22% | 25% | Heterozygous | GG vs. AG | 0.444 | 1.450 | ( | 0.558 | - | 3.767 | ) |
|  |  |  |  |  |  |  |  |  |  |  |  |  |  |
| rs2227982 | 241851281 | AA | AG | GG | Additive | AA vs.AG vs. GG | 0.505 | NA |  |  |  |  |  |
| RF+ | PDCD1 | 27 | 35 | 23 | Dominant | AA vs. AG+GG | 0.602 | 0.781 | ( | 0.308 | - | 1.978 | ) |
|  |  | 77% | 69% | 79% | Recessive | AA+AG vs. GG | 0.444 | 1.484 | ( | 0.538 | - | 4.093 | ) |
| RF- |  | 8 | 16 | 6 | Homozygous | AA vs. GG | 0.835 | 1.136 | ( | 0.344 | - | 3.755 | ) |
|  |  | 23% | 31% | 21% | Heterozygous | AA vs. AG | 0.387 | 0.648 | ( | 0.242 | - | 1.737 | ) |
|  |  |  |  |  |  |  |  |  |  |  |  |  |  |
| rs6705653 | 241851407 | CC | CT | TT | Additive | CC vs. CT vs. TT | 0.839 | NA |  |  |  |  |  |
| RF+ | PDCD1 | 48 | 29 | 10 | Dominant | CC vs. CT+TT | 0.555 | 1.286 | ( | 0.557 | - | 2.971 | ) |
|  |  | 72% | 76% | 77% | Recessive | CC+CT vs. TT | 1.000 | 1.212 | ( | 0.311 | - | 4.726 | ) |
| RF- |  | 19 | 9 | 3 | Homozygous | CC vs. TT | 1.000 | 1.319 | ( | 0.327 | - | 5.325 | ) |
|  |  | 28% | 24% | 23% | Heterozygous | CC vs. CT | 0.603 | 1.275 | ( | 0.510 | - | 3.192 | ) |
|  |  |  |  |  |  |  |  |  |  |  |  |  |  |
| rs41386349 | 241851697 | GG | AG | AA | Additive | GG vs. AG vs. AA | 0.833 | NA |  |  |  |  |  |
| RF+ | PDCD1 | 52 | 27 | 7 | Dominant | GG vs. AG+AA | 0.547 | 1.308 | ( | 0.546 | - | 3.132 | ) |
|  |  | 72% | 77% | 78% | Recessive | GG+AG vs. AA | 1.000 | 1.241 | ( | 0.243 | - | 6.328 | ) |
| RF- |  | 20 | 8 | 2 | Homozygous | GG vs. AA | 1.000 | 1.346 | ( | 0.258 | - | 7.037 | ) |
|  |  | 28% | 23% | 22% | Heterozygous | GG vs. AG | 0.587 | 1.298 | ( | 0.506 | - | 3.332 | ) |
|  |  |  |  |  |  |  |  |  |  |  |  |  |  |
| rs36084323 | 241859444 | TT | CT | CC | Additive | TT vs. CT vs.CC | 0.420 | NA |  |  |  |  |  |
| RF+ |  | 23 | 44 | 25 | Dominant | TT vs. CT+CC | 0.725 | 1.174 | ( | 0.476 | - | 2.898 | ) |
|  |  | 72% | 71% | 83% | Recessive | TT+CT vs. CC | 0.189 | 2.015 | ( | 0.699 | - | 5.810 | ) |
| RF- |  | 9 | 18 | 5 | Homozygous | TT vs. CC | 0.281 | 1.957 | ( | 0.571 | - | 6.702 | ) |
|  |  | 28% | 29% | 17% | Heterozygous | TT vs. CT | 0.927 | 0.957 | ( | 0.371 | - | 2.463 | ) |
|  |  |  |  |  |  |  |  |  |  |  |  |  |  |
| rs5839828 | 241859601 | DEL | DEL/G | GG | Additive | DEL vs. DEL/G vs.GG | 0.575 | NA |  |  |  |  |  |
| RF+ |  | 31 | 46 | 15 | Dominant | DEL vs. DEL/G+GG | 0.480 | 1.346 | ( | 0.589 | - | 3.080 | ) |
|  |  | 71% | 74% | 83% | Recessive | DEL+DEL/G vs.GG |  |  |  |  |  |  | ) |
| RF- |  | 13 | 16 | 3 | Homozygous | DEL vs.GG | 0.355 | 2.097 | ( | 0.518 | - | 8.491 | ) |
|  |  | 29% | 26% | 17% | Heterozygous | DEL vs. DEL/G | 0.670 | 1.206 | ( | 0.509 | - | 2.855 | ) |
| RF: rheumatoid factor; Additive: AA vs. Aa vs. aa; Dominant: AA vs. Aa+aa; Recessive: AA+Aa vs. aa; Homozygous: AA vs. aa; Heterozygous: AA vs. Aa, where the frequency of A-allele is major in the population, and a-allele is minor. *:<0.05; NA: not applicable. | | | | | | | | | | | | | |

**Supplementary Table 6 The association between SNPs and anti-CCP.**

| **SNP** | **Gene position** | **No. of patients (%)** | | | **Model** | | **Logistic regression p** | **OR (95 % CI)** | | | | | |
| --- | --- | --- | --- | --- | --- | --- | --- | --- | --- | --- | --- | --- | --- |
| rs181758110 | 173208023 | GG | GA | AA | Additive | GG vs. GA vs. AA | NA | NA |  |  |  |  |  |
| ACCP+ | TNFSF4 | 20 | 0 | 0 | Dominant | GG vs. GA+AA | NA | NA |  |  |  |  |  |
|  |  | 16% | 0% | 0% | Recessive | GG+GA vs. AA | NA | NA |  |  |  |  |  |
| ACCP- |  | 104 | 0 | 0 | Homozygous | GG vs. AA | NA | NA |  |  |  |  |  |
|  |  | 84% | 0% | 0% | Heterozygous | GG vs. GA | NA | NA |  |  |  |  |  |
|  |  |  |  |  |  |  |  |  |  |  |  |  |  |
| rs45454293 | 173208097 | CC | CT | TT | Additive | CC vs. CT vs. TT | 0.244 | NA |  |  |  |  |  |
| ACCP+ | TNFSF4 | 13 | 7 | 0 | Dominant | CC vs. CT+TT | 0.354 | 1.615 | ( | 0.582 | - | 4.482 | ) |
|  |  | 14% | 25% | 0% | Recessive | CC+CT vs. TT | 0.592 | NA |  |  |  |  |  |
| ACCP- |  | 78 | 21 | 5 | Homozygous | CC vs. TT | 1.000 | NA |  |  |  |  |  |
|  |  | 86% | 75% | 100% | Heterozygous | CC vs. CT | 0.246 | 2.000 | ( | 0.709 | - | 5.644 | ) |
|  |  |  |  |  |  |  |  |  |  |  |  |  |  |
| rs1234314 | 173208253 | CC | CG | GG | Additive | CC vs.CG vs. GG | 0.467 | NA |  |  |  |  |  |
| ACCP+ | TNFSF4 | 6 | 11 | 3 | Dominant | CC vs. CG+GG | 0.304 | 1.711 | ( | 0.609 | - | 4.805 | ) |
|  |  | 12% | 21% | 14% | Recessive | CC+CG vs. GG | 1.000 | 0.843 | ( | 0.223 | - | 3.183 | ) |
| ACCP- |  | 44 | 42 | 18 | Homozygous | CC vs. GG | 1.000 | 1.222 | ( | 0.275 | - | 5.426 | ) |
|  |  | 88% | 79% | 86% | Heterozygous | CC vs. CG | 0.232 | 1.921 | ( | 0.652 | - | 5.660 | ) |
|  |  |  |  |  |  |  |  |  |  |  |  |  |  |
| rs1879877 | 203705277 | TT | GT | GG | Additive | TT vs.GT vs.GG | 0.400 | NA |  |  |  |  |  |
| ACCP+ | CD28 | 9 | 10 | 1 | Dominant | TT vs. GT+GG | 0.475 | 0.704 | ( | 0.268 | - | 1.851 | ) |
|  |  | 19% | 17% | 6% | Recessive | GG vs. TT+GT | 0.302 | 0.269 | ( | 0.034 | - | 2.149 | ) |
| ACCP- |  | 38 | 49 | 17 | Homozygous | TT vs. GG | 0.261 | 0.248 | ( | 0.029 | - | 2.119 | ) |
|  |  | 81% | 83% | 94% | Heterozygous | TT vs. GT | 0.769 | 0.862 | ( | 0.319 | - | 2.331 | ) |
|  |  |  |  |  |  |  |  |  |  |  |  |  |  |
| rs3181096 | 203705369 | CC | CT | TT | Additive | CC vs. CT vs. TT | 0.500 | NA |  |  |  |  |  |
| ACCP+ | CD28 | 14 | 6 | 0 | Dominant | CC vs. CT+TT | 0.342 | 0.608 | ( | 0.216 | - | 1.708 | ) |
|  |  | 19% | 13% | 0% | Recessive | CC+CT vs. TT | 1.000 | NA |  |  |  |  | ) |
| ACCP- |  | 61 | 39 | 4 | Homozygous | CC vs. TT | 1.000 | NA |  |  |  |  | ) |
|  |  | 81% | 87% | 100% | Heterozygous | CC vs. CT | 0.448 | 0.670 | ( | 0.238 | - | 1.891 | ) |
|  |  |  |  |  |  |  |  |  |  |  |  |  |  |
| rs3181097 | 203705416 | GG | AG | AA | Additive | GG vs. AG vs. AA | 0.902 | NA |  |  |  |  |  |
| ACCP+ | CD28 | 5 | 12 | 3 | Dominant | GG vs. AG+AA | 0.859 | 1.105 | ( | 0.368 | - | 3.324 | ) |
|  |  | 15% | 17% | 14% | Recessive | GG +AG vs.AA | 1.000 | 0.789 | ( | 0.210 | - | 2.968 | ) |
| ACCP- |  | 28 | 57 | 19 | Homozygous | GG vs. AA | 1.000 | 0.884 | ( | 0.189 | - | 4.147 | ) |
|  |  | 85% | 83% | 86% | Heterozygous | GG vs. AG | 0.776 | 1.176 | ( | 0.378 | - | 3.676 | ) |
|  |  |  |  |  |  |  |  |  |  |  |  |  |  |
| rs3181098 | 203705655 | GG | AG | AA | Additive | GG vs. AG vs. AA | 0.560 | NA |  |  |  |  |  |
| ACCP+ | CD28 | 14 | 6 | 0 | Dominant | GG vs. AG+AA | 0.426 | 0.659 | ( | 0.234 | - | 1.852 | ) |
|  |  | 18% | 14% | 0% | Recessive | GG+AG vs. AA | 1.000 | NA |  |  |  |  | ) |
| ACCP- |  | 63 | 37 | 4 | Homozygous | GG vs. AA | 1.000 | NA |  |  |  |  | ) |
|  |  | 82% | 86% | 100% | Heterozygous | GG vs. AG | 0.551 | 0.730 | ( | 0.258 | - | 2.062 | ) |
|  |  |  |  |  |  |  |  |  |  |  |  |  |  |
| rs28688913 | 203705805 | CC | CT | TT | Additive | CC vs. CT vs. TT | 0.741 | NA |  |  |  |  |  |
| ACCP+ | CD28 | 15 | 4 | 1 | Dominant | CC vs. CT+TT | 0.664 | 0.785 | ( | 0.262 | - | 2.348 | ) |
|  |  | 17% | 13% | 25% | Recessive | CC+CT vs. TT | 0.510 | 1.772 | ( | 0.175 | - | 17.952 | ) |
| ACCP- |  | 73 | 28 | 3 | Homozygous | CC vs. TT | 0.541 | 1.622 | ( | 0.158 | - | 16.679 | ) |
|  |  | 83% | 87% | 75% | Heterozygous | CC vs. CT | 0.546 | 0.695 | ( | 0.212 | - | 2.276 | ) |
|  |  |  |  |  |  |  |  |  |  |  |  |  |  |
| rs56228674 | 203729436 | CC | CT | TT | Additive | CC vs. CT vs. TT | 0.475 | 1.750 | ( | 0.506 | - | 6.049 | ) |
| ACCP+ | CD28 | 16 | 4 | 0 | Dominant | CC vs. CT+TT | 0.475 | 1.750 | ( | 0.506 | - | 6.049 | ) |
|  |  | 15% | 24% | 0% | Recessive | CC+CT vs. TT | NA | NA |  |  |  |  |  |
| ACCP- |  | 91 | 13 | 0 | Homozygous | CC vs. TT | NA | NA |  |  |  |  |  |
|  |  | 85% | 76% | 0% | Heterozygous | CC vs. CT | 0.475 | 1.750 | ( | 0.506 | - | 6.049 | ) |
|  |  |  |  |  |  |  |  |  |  |  |  |  |  |
| rs3116496 | 203729789 | TT | CT | CC | Additive | TT vs. CT vs.CC | 0.633 | NA |  |  |  |  |  |
| ACCP+ | CD28 | 15 | 4 | 1 | Dominant | TT vs. CT+CC | 0.791 | 0.862 | ( | 0.287 | - | 2.588 | ) |
|  |  | 17% | 13% | 33% | Recessive | TT+CT vs. CC | 0.413 | 2.684 | ( | 0.232 | - | 31.103 | ) |
| ACCP- |  | 75 | 27 | 2 | Homozygous | TT vs. CC | 0.436 | 2.500 | ( | 0.213 | - | 29.369 | ) |
|  |  | 83% | 87% | 67% | Heterozygous | TT vs. CT | 0.778 | 0.741 | ( | 0.226 | - | 2.429 | ) |
|  |  |  |  |  |  |  |  |  |  |  |  |  |  |
| rs11571315 | 203866178 | TT | CT | CC | Additive | TT vs.TC vs.CC | 0.391 | NA |  |  |  |  |  |
| ACCP+ | CTLA4 | 10 | 8 | 2 | Dominant | TT vs. TC+CC | 0.428 | 1.475 | ( | 0.562 | - | 3.868 | ) |
|  |  | 15% | 24% | 12% | Recessive | TT+TC vs. CC | 0.734 | 0.622 | ( | 0.131 | - | 2.963 | ) |
| ACCP- |  | 59 | 25 | 15 | Homozygous | TT vs. CC | 1.000 | 0.787 | ( | 0.156 | - | 3.977 | ) |
|  |  | 85% | 76% | 88% | Heterozygous | TT vs. TC | 0.277 | 1.888 | ( | 0.667 | - | 5.345 | ) |
|  |  |  |  |  |  |  |  |  |  |  |  |  |  |
| rs733618 | 203866221 | TT | CT | CC | Additive | TT vs.TC vs.CC | 0.677 | NA |  |  |  |  |  |
| ACCP+ | CTLA4 | 6 | 9 | 5 | Dominant | TT vs. TC+CC | 0.688 | 1.237 | ( | 0.437 | - | 3.502 | ) |
|  |  | 15% | 21% | 14% | Recessive | TT+TC vs. CC | 0.611 | 0.753 | ( | 0.251 | - | 2.254 | ) |
| ACCP- |  | 35 | 35 | 31 | Homozygous | TT vs. CC | 0.926 | 0.941 | ( | 0.261 | - | 3.389 | ) |
|  |  | 85% | 79% | 86% | Heterozygous | TT vs. TC | 0.482 | 1.500 | ( | 0.482 | - | 4.663 | ) |
|  |  |  |  |  |  |  |  |  |  |  |  |  |  |
| rs4553808 | 203866221 | AA | AG | GG | Additive | AA vs.AG vs. GG | 0.424 |  |  |  |  |  |  |
| ACCP+ | CTLA4 | 16 | 4 | 0 | Dominant | AA vs. AG+GG | 0.740 | 1.359 | ( | 0.402 | - | 4.597 | ) |
|  |  | 16% | 25% | 0% | Recessive | AA+AG vs. GG | 1.000 | NA |  |  |  |  |  |
| ACCP- |  | 87 | 12 | 4 | Homozygous | AA vs. GG | 1.000 | NA |  |  |  |  |  |
|  |  | 85% | 75% | 100% | Heterozygous | AA vs. AG | 0.470 | 1.813 | ( | 0.519 | - | 6.332 | ) |
|  |  |  |  |  |  |  |  |  |  |  |  |  |  |
| rs11571316 | 203866366 | GG | AG | AA | Additive | GG vs. AG vs. AA | 0.126 | NA |  |  |  |  |  |
| ACCP+ | CTLA4 | 13 | 7 | 0 | Dominant | GG vs. AG+AA | 0.317 | 1.680 | ( | 0.604 | - | 4.674 | ) |
|  |  | 14% | 28% | 0% | Recessive | GG+AG vs. AA | 0.597 | NA |  |  |  |  | ) |
| ACCP- |  | 78 | 18 | 7 | Homozygous | GG vs. AA | 0.589 | NA |  |  |  |  | ) |
|  |  | 86% | 72% | 100% | Heterozygous | GG vs. AG | 0.135 | 2.333 | ( | 0.815 | - | 6.682 | ) |
|  |  |  |  |  |  |  |  |  |  |  |  |  |  |
| rs62182595 | 203866465 | GG | AG | AA | Additive | GG vs. AG vs. AA | 0.487 | NA |  |  |  |  |  |
| ACCP+ | CTLA4 | 16 | 4 | 0 | Dominant | GG vs. AG+AA | 0.748 | 1.250 | ( | 0.372 | - | 4.205 | ) |
|  |  | 16% | 24% | 0% | Recessive | GG+AG vs. AA | 1.000 | NA |  |  |  |  | ) |
| ACCP- |  | 85 | 13 | 4 | Homozygous | GG vs. AA | 1.000 | NA |  |  |  |  | ) |
|  |  | 84% | 76% | 100% | Heterozygous | GG vs. AG | 0.485 | 1.635 | ( | 0.472 | - | 5.657 | ) |
|  |  |  |  |  |  |  |  |  |  |  |  |  |  |
| rs16840252 | 203866796 | CC | CT | TT | Additive | CC vs. CT vs. TT | 0.299 | NA |  |  |  |  |  |
| ACCP+ | CTLA4 | 16 | 4 | 0 | Dominant | CC vs. CT+TT | 0.491 | 1.589 | ( | 0.464 | - | 5.449 | ) |
|  |  | 15% | 29% | 16% | Recessive | CC+CT vs. TT | 1.000 | NA |  |  |  |  |  |
| ACCP- |  | 89 | 10 | 4 | Homozygous | CC vs. TT | 1.000 | NA |  |  |  |  |  |
|  |  | 85% | 71% | 84% | Heterozygous | CC vs. CT | 0.251 | 2.225 | ( | 0.621 | - | 7.969 | ) |
|  |  |  |  |  |  |  |  |  |  |  |  |  |  |
| rs5742909 | 203867624 | CC | CT | TT | Additive | CC vs. CT vs. TT | 0.506 | NA |  |  |  |  |  |
| ACCP+ | CTLA4 | 15 | 5 | 0 | Dominant | CC vs. CT+TT | 1.000 | 1.111 | ( | 0.366 | - | 3.372 | ) |
|  |  | 16% | 21% | 0% | Recessive | CC+CT vs. TT | 0.592 | NA |  |  |  |  |  |
| ACCP- |  | 80 | 19 | 5 | Homozygous | CC vs. TT | 1.000 | NA |  |  |  |  |  |
|  |  | 84% | 79% | 100% | Heterozygous | CC vs. CT | 0.550 | 1.404 | ( | 0.454 | - | 4.340 | ) |
|  |  |  |  |  |  |  |  |  |  |  |  |  |  |
| rs231775 | 203867991 | GG | AG | AA | Additive | GG vs. AG vs. AA | 0.756 | NA |  |  |  |  |  |
| ACCP+ | CTLA4 | 10 | 8 | 2 | Dominant | GG vs. AG+AA | 1.000 | 1.000 | ( | 0.381 | - | 2.626 | ) |
|  |  | 18% | 20% | 12% | Recessive | GG+AG vs. AA | 0.733 | 0.585 | ( | 0.123 | - | 2.789 | ) |
| ACCP- |  | 47 | 32 | 15 | Homozygous | GG vs. AA | 0.721 | 0.627 | ( | 0.123 | - | 3.185 | ) |
|  |  | 82% | 80% | 88% | Heterozygous | GG vs. AG | 0.795 | 1.175 | ( | 0.418 | - | 3.300 | ) |
|  |  |  |  |  |  |  |  |  |  |  |  |  |  |
| rs3087243 | 203874196 | GG | AG | AA | Additive | GG vs. AG vs. AA | 0.367 | NA |  |  |  |  |  |
| ACCP+ | CTLA4 | 13 | 7 | 0 | Dominant | GG vs. AG+AA | 0.775 | 1.159 | ( | 0.423 | - | 3.172 | ) |
|  |  | 16% | 21% | 0% | Recessive | GG+AG vs. AA | 0.597 | NA |  |  |  |  | ) |
| ACCP- |  | 71 | 26 | 7 | Homozygous | GG vs. AA | 0.587 | NA |  |  |  |  | ) |
|  |  | 84% | 79% | 100% | Heterozygous | GG vs. AG | 0.458 | 1.470 | ( | 0.529 | - | 4.089 | ) |
|  |  |  |  |  |  |  |  |  |  |  |  |  |  |
| rs11571319 | 203874215 | GG | AG | AA | Additive | GG vs. GA vs. AA | 0.242 |  |  |  |  |  |  |
| ACCP+ | CTLA4 | 14 | 5 | 1 | Dominant | GG vs. GA+AA | 0.708 | 1.222 | ( | 0.427 | - | 3.500 | ) |
|  |  | 15% | 28% | 7% | Recessive | GG+GA vs. AA | 0.462 | 0.338 | ( | 0.042 | - | 2.731 | ) |
| ACCP- |  | 77 | 13 | 14 | Homozygous | GG vs. AA | 0.690 | 0.393 | ( | 0.048 | - | 3.231 | ) |
|  |  | 85% | 72% | 93% | Heterozygous | GG vs. GA | 0.304 | 2.115 | ( | 0.651 | - | 6.872 | ) |
|  |  |  |  |  |  |  |  |  |  |  |  |  |  |
| rs10204525 | 241850169 | TT | CT | CC | Additive | TT vs. CT vs.CC | 0.581 | NA |  |  |  |  |  |
| ACCP+ | PDCD1 | 11 | 9 | 0 | Dominant | TT vs. CT+CC | 0.625 | 0.787 | ( | 0.301 | - | 2.059 | ) |
|  |  | 18% | 16% | 0% | Recessive | TT+CT vs. CC | 0.592 | NA |  |  |  |  | ) |
| ACCP- |  | 51 | 48 | 5 | Homozygous | TT vs. CC | 0.582 | NA |  |  |  |  | ) |
|  |  | 82% | 84% | 100% | Heterozygous | TT vs. CT | 0.776 | 0.869 | ( | 0.331 | - | 2.282 | ) |
|  |  |  |  |  |  |  |  |  |  |  |  |  |  |
| rs56029561 | 241850737 | DEL | DEL/CAG | CAG | Additive | DEL vs. DEL/CAG vs.CAG | 1.000 | 1.176 | ( | 0.233 | - | 5.930 | ) |
| ACCP+ | PDCD1 | 17 | 2 | 0 | Dominant | DEL vs.DEL/CAG+CAG | 1.000 | 1.176 | ( | 0.233 | - | 5.930 | ) |
|  |  | 16% | 18% | 0% | Recessive | DEL+DEL/CAG vs.CAG | NA | NA |  |  |  |  |  |
| ACCP- |  | 90 | 9 | 0 | Homozygous | DEL vs.CAG | NA | NA |  |  |  |  |  |
|  |  | 84% | 82% | 0% | Heterozygous | DEL vs.DEL/CAG | 1.000 | 1.176 | ( | 0.233 | - | 5.930 | ) |
|  |  |  |  |  |  |  |  |  |  |  |  |  |  |
| rs2227981 | 241851121 | GG | AG | AA | Additive | GG vs. AG vs. AA | 0.161 | NA |  |  |  |  |  |
| ACCP+ | PDCD1 | 6 | 8 | 3 | Dominant | GG vs. AG+AA | 0.059 | 2.750 | ( | 0.938 | - | 8.066 | ) |
|  |  | 9% | 22% | 25% | Recessive | GG+AG vs. AA | 0.388 | 2.048 | ( | 0.493 | - | 8.501 | ) |
| ACCP- |  | 57 | 29 | 9 | Homozygous | GG vs. AA | 0.151 | 3.167 | ( | 0.669 | - | 14.980 | ) |
|  |  | 91% | 78% | 75% | Heterozygous | GG vs. AG | 0.092 | 2.621 | ( | 0.831 | - | 8.269 | ) |
|  |  |  |  |  |  |  |  |  |  |  |  |  |  |
| rs2227982 | 241851281 | AA | AG | GG | Additive | AA vs.AG vs. GG | 0.370 | NA |  |  |  |  |  |
| ACCP+ | PDCD1 | 3 | 10 | 5 | Dominant | AA vs. AG+GG | 0.167 | 2.462 | ( | 0.664 | - | 9.122 | ) |
|  |  | 9% | 20% | 17% | Recessive | AA+AG vs. GG | 0.773 | 1.170 | ( | 0.378 | - | 3.621 | ) |
| ACCP- |  | 32 | 41 | 24 | Homozygous | AA vs. GG | 0.451 | 2.222 | ( | 0.483 | - | 10.222 | ) |
|  |  | 91% | 80% | 83% | Heterozygous | AA vs. AG | 0.160 | 2.602 | ( | 0.661 | - | 10.244 | ) |
|  |  |  |  |  |  |  |  |  |  |  |  |  |  |
| rs6705653 | 241851407 | CC | CT | TT | Additive | CC vs. CT vs. TT | 0.160 | NA |  |  |  |  |  |
| ACCP+ | PDCD1 | 7 | 9 | 3 | Dominant | CC vs. CT+TT | 0.055 | 2.637 | ( | 0.955 | - | 7.282 | ) |
|  |  | 10% | 24% | 23% | Recessive | CC+CT vs. TT | 0.438 | 1.669 | ( | 0.413 | - | 6.738 | ) |
| ACCP- |  | 60 | 29 | 10 | Homozygous | CC vs. TT | 0.353 | 2.571 | ( | 0.569 | - | 11.630 | ) |
|  |  | 90% | 76% | 77% | Heterozygous | CC vs. CT | 0.070 | 2.660 | ( | 0.901 | - | 7.854 | ) |
|  |  |  |  |  |  |  |  |  |  |  |  |  |  |
| rs41386349 | 241851697 | GG | AG | AA | Additive | GG vs. AG vs. AA | 0.110 | NA |  |  |  |  |  |
| ACCP+ | PDCD1 | 8 | 8 | 3 | Dominant | GG vs. AG+AA | 0.050* | 2.667 | ( | 0.978 | - | 7.270 | ) |
|  |  | 11% | 23% | 33% | Recessive | GG+AG vs. AA | 0.164 | 2.844 | ( | 0.645 | - | 12.546 | ) |
| ACCP- |  | 64 | 27 | 6 | Homozygous | GG vs. AA | 0.100 | 4.000 | ( | 0.833 | - | 19.202 | ) |
|  |  | 89% | 77% | 67% | Heterozygous | GG vs. AG | 0.110 | 2.370 | ( | 0.806 | - | 6.968 | ) |
|  |  |  |  |  |  |  |  |  |  |  |  |  |  |
| rs36084323 | 241859444 | TT | CT | CC | Additive | TT vs. CT vs.CC | 0.725 | NA |  |  |  |  |  |
| ACCP+ |  | 4 | 10 | 6 | Dominant | TT vs. CT+CC | 0.517 | 1.474 | ( | 0.454 | - | 4.787 | ) |
|  |  | 13% | 16% | 20% | Recessive | TT+CT vs. CC | 0.570 | 1.429 | ( | 0.495 | - | 4.122 | ) |
| ACCP- |  | 28 | 52 | 24 | Homozygous | TT vs. CC | 0.502 | 1.750 | ( | 0.441 | - | 6.940 | ) |
|  |  | 87% | 84% | 80% | Heterozygous | TT vs. CT | 0.766 | 1.346 | ( | 0.387 | - | 4.666 | ) |
|  |  |  |  |  |  |  |  |  |  |  |  |  |  |
| rs5839828 | 241859601 | DEL | DEL/G | GG | Additive | DEL vs. DEL/G vs.GG | 0.580 | NA |  |  |  |  |  |
| ACCP+ |  | 9 | 8 | 3 | Dominant | DEL vs. DEL/G+GG | 0.331 | 0.620 | ( | 0.235 | - | 1.636 | ) |
|  |  | 21% | 13% | 17% | Recessive | DEL+DEL/G vs.GG | 1.000 | 1.047 | ( | 0.273 | - | 4.014 | ) |
| ACCP- |  | 35 | 54 | 15 | Homozygous | DEL vs.GG | 1.000 | 0.778 | ( | 0.184 | - | 3.282 | ) |
|  |  | 79% | 87% | 83% | Heterozygous | DEL vs. DEL/G | 0.297 | 0.576 | ( | 0.203 | - | 1.635 | ) |
| ACCP: anti-cyclic citrullinated peptide antibody; Additive: AA vs. Aa vs. aa; Dominant: AA vs. Aa+aa; Recessive: AA+Aa vs. aa; Homozygous: AA vs. aa; Heterozygous: AA vs. Aa, where the frequency of A-allele is major in the population, and a-allele is minor. *:<0.05; NA: not applicable.*: P<0.05; NA: not applicable | | | | | | | | | | | | | |

**Supplementary Table 7. The association between SNPs and CRP.**

| **SNP** | **Gene position** | **No. of patients (%)** | | | **Model** | | **Logistic regression p** | **OR (95 % CI)** | | | | | |
| --- | --- | --- | --- | --- | --- | --- | --- | --- | --- | --- | --- | --- | --- |
| rs181758110 | 173208023 | GG | GA | AA | Additive | GG vs. GA vs. AA | NA | NA |  |  |  |  |  |
| CRP+ | TNFSF4 | 75 | 0 | 0 | Dominant | GG vs. GA+AA | NA | NA |  |  |  |  |  |
|  |  | 61% | 0% | 0% | Recessive | GG+GA vs. AA | NA | NA |  |  |  |  |  |
| CRP- |  | 49 | 0 | 0 | Homozygous | GG vs. AA | NA | NA |  |  |  |  |  |
|  |  | 39% | 0% | 0% | Heterozygous | GG vs. GA | NA | NA |  |  |  |  |  |
|  |  |  |  |  |  |  |  |  |  |  |  |  |  |
| rs45454293 | 173208097 | CC | CT | TT | Additive | CC vs. CT vs. TT | 0.553 | NA |  |  |  |  |  |
| CRP+ | TNFSF4 | 57 | 16 | 2 | Dominant | CC vs. CT+TT | 0.415 | 0.716 | ( | 0.320 | - | 1.6030 | ) |
|  |  | 63% | 57% | 40% | Recessive | CC+CT vs. TT | 0.383 | 0.420 | ( | 0.068 | - | 2.611 | ) |
| CRP- |  | 34 | 12 | 3 | Homozygous | CC vs. TT | 0.370 | 0.398 | ( | 0.063 | - | 2.501 | ) |
|  |  | 37% | 43% | 60% | Heterozygous | CC vs. CT | 0.602 | 0.795 | ( | 0.336 | - | 1.881 | ) |
|  |  |  |  |  |  |  |  |  |  |  |  |  |  |
| rs1234314 | 173208253 | CC | CG | GG | Additive | CC vs.CG vs. GG | 0.352 | NA |  |  |  |  |  |
| CRP+ | TNFSF4 | 33 | 32 | 10 | Dominant | CC vs. CG+GG | 0.302 | 0.676 | ( | 0.321 | - | 1.423 | ) |
|  |  | 66% | 60% | 48% | Recessive | CC+CG vs. GG | 0.186 | 0.531 | ( | 0.207 | - | 1.368 | ) |
| CRP- |  | 17 | 21 | 11 | Homozygous | CC vs. GG | 0.148 | 0.468 | ( | 0.166 | - | 1.321 | ) |
|  |  | 34% | 40% | 52% | Heterozygous | CC vs. CG | 0.554 | 0.785 | ( | 0.352 | - | 1.753 | ) |
|  |  |  |  |  |  |  |  |  |  |  |  |  |  |
| rs1879877 | 203705277 | TT | GT | GG | Additive | TT vs.GT vs.GG | 0.804 | NA |  |  |  |  |  |
| CRP+ | CD28 | 30 | 35 | 10 | Dominant | TT vs. GT+GG | 0.644 | 0.788 | ( | 0.288 | - | 2.162 | ) |
|  |  | 64% | 59% | 56% | Recessive | GG vs. TT+GT | 0.552 | 0.797 | ( | 0.377 | - | 1.684 | ) |
| CRP- |  | 17 | 24 | 8 | Homozygous | TT vs. GG | 0.539 | 0.708 | ( | 0.235 | - | 2.136 | ) |
|  |  | 36% | 41% | 44% | Heterozygous | TT vs. GT | 0.636 | 0.826 | ( | 0.375 | - | 1.821 | ) |
|  |  |  |  |  |  |  |  |  |  |  |  |  |  |
| rs3181096 | 203705369 | CC | CT | TT | Additive | CC vs. CT vs. TT | 0.022* | NA |  |  |  |  |  |
| CRP+ | CD28 | 52 | 20 | 3 | Dominant | CC vs. CT+TT | 0.013* | 0.391 | ( | 0.186 | - | 0.825 | ) |
|  |  | 69% | 44% | 75% | Recessive | CC+CT vs. TT | 1.000 | 2.000 | ( | 0.202 | - | 19.798 | ) |
| CRP- |  | 23 | 25 | 1 | Homozygous | CC vs. TT | 1.000 | 1.327 | ( | 0.131 | - | 13.445 | ) |
|  |  | 31% | 56% | 25% | Heterozygous | CC vs. CT | 0.007* | 0.354 | ( | 0.165 | - | 0.7611 | ) |
|  |  |  |  |  |  |  |  |  |  |  |  |  |  |
| rs3181097 | 203705416 | GG | AG | AA | Additive | GG vs. AG vs. AA | 0.891 | NA |  |  |  |  |  |
| CRP+ | CD28 | 19 | 43 | 13 | Dominant | GG vs. AG+AA | 0.690 | 1.179 | ( | 0.525 | - | 2.648 | ) |
|  |  | 58% | 62% | 59% | Recessive | GG +AG vs.AA | 0.883 | 0.932 | ( | 0.365 | - | 2.381 | ) |
| CRP- |  | 9 | 26 | 14 | Homozygous | GG vs. AA | 0.911 | 1.064 | ( | 0.356 | - | 3.181 | ) |
|  |  | 42% | 38% | 41% | Heterozygous | GG vs. AG | 0.646 | 1.219 | ( | 0.524 | - | 2.836 | ) |
|  |  |  |  |  |  |  |  |  |  |  |  |  |  |
| rs3181098 | 203705655 | GG | AG | AA | Additive | GG vs. AG vs. AA | 0.008* | NA |  |  |  |  |  |
| CRP+ | CD28 | 54 | 18 | 3 | Dominant | GG vs. AG+AA | 0.005* | 0.344 | ( | 0.162 | - | 0.731 | ) |
|  |  | 70% | 42% | 75% | Recessive | GG+AG vs. AA | 1.000 | 2.000 | ( | 0.202 | - | 19.798 | ) |
| CRP- |  | 23 | 25 | 1 | Homozygous | GG vs. AA | 1.000 | 1.278 | ( | 0.126 | - | 12.940 | ) |
|  |  | 30% | 58% | 25% | Heterozygous | GG vs. AG | 0.002* | 0.307 | ( | 0.141 | - | 0.668 | ) |
|  |  |  |  |  |  |  |  |  |  |  |  |  |  |
| rs28688913 | 203705805 | CC | CT | TT | Additive | CC vs. CT vs. TT | 0.107 | NA |  |  |  |  |  |
| CRP+ | CD28 | 48 | 24 | 3 | Dominant | CC vs. CT+TT | 0.034* | 2.500 | ( | 1.054 | - | 5.927 | ) |
|  |  | 55% | 75% | 75% | Recessive | CC+CT vs. TT | 1.000 | 2.000 | ( | 0.202 | - | 19.798 | ) |
| CRP- |  | 40 | 8 | 1 | Homozygous | CC vs. TT | 0.626 | 2.500 | ( | 0.250 | - | 24.979 | ) |
|  |  | 45% | 25% | 25% | Heterozygous | CC vs. CT | 0.043* | 2.500 | ( | 1.013 | - | 6.171 | ) |
|  |  |  |  |  |  |  |  |  |  |  |  |  |  |
| rs56228674 | 203729436 | CC | CT | TT | Additive | CC vs. CT vs. TT | 0.880 | 0.923 | ( | 0.326 | - | 2.614 | ) |
| CRP+ | CD28 | 65 | 10 | 0 | Dominant | CC vs. CT+TT | 0.880 | 0.923 | ( | 0.326 | - | 2.614 | ) |
|  |  | 61% | 59% | 0% | Recessive | CC+CT vs. TT | NA | NA |  |  |  |  |  |
| CRP- |  | 42 | 7 | 0 | Homozygous | CC vs. TT | NA | NA |  |  |  |  |  |
|  |  | 59% | 61% | 0% | Heterozygous | CC vs. CT | 0.880 | 0.923 | ( | 0.326 | - | 2.614 | ) |
|  |  |  |  |  |  |  |  |  |  |  |  |  |  |
| rs3116496 | 203729789 | TT | CT | CC | Additive | TT vs. CT vs.CC | 0.968 | NA |  |  |  |  |  |
| CRP+ | CD28 | 54 | 19 | 2 | Dominant | TT vs. CT+CC | 0.858 | 1.077 | ( | 0.479 | - | 2.421 | ) |
|  |  | 60% | 61% | 67% | Recessive | TT+CT vs. CC | 1.000 | 1..315 | ( | 0.116 | - | 14.907 | ) |
| CRP- |  | 36 | 12 | 1 | Homozygous | TT vs. CC | 1.000 | 1.333 | ( | 0.117 | - | 15.255 | ) |
|  |  | 40% | 39% | 33% | Heterozygous | TT vs. CT | 0.899 | 1.056 | ( | 0.457 | - | 2.437 | ) |
|  |  |  |  |  |  |  |  |  |  |  |  |  |  |
| rs11571315 | 203866178 | TT | CT | CC | Additive | TT vs.TC vs.CC | 0.426 | NA |  |  |  |  |  |
| CRP+ | CTLA4 | 44 | 21 | 8 | Dominant | TT vs. TC+CC | 0.524 | 0.785 | ( | 0.372 | - | 1.654 | ) |
|  |  | 64% | 64% | 47% | Recessive | TT+TC vs. CC | 0.191 | 0.506 | ( | 0.180 | - | 1.424 | ) |
| CRP- |  | 25 | 12 | 9 | Homozygous | TT vs. CC | 0.207 | 0.505 | ( | 0.173 | - | 1.475 | ) |
|  |  | 36% | 36% | 53% | Heterozygous | TT vs. TC | 0.990 | 0.994 | ( | 0.420 | - | 2.356 | ) |
|  |  |  |  |  |  |  |  |  |  |  |  |  |  |
| rs733618 | 203866221 | TT | CT | CC | Additive | TT vs.TC vs.CC | 0.017* | NA |  |  |  |  |  |
| CRP+ | CTLA4 | 23 | 34 | 17 | Dominant | TT vs. TC+CC | 0.414 | 1.376 | ( | 0.639 | - | 2.963 | ) |
|  |  | 56% | 77% | 47% | Recessive | TT+TC vs. CC | 0.041* | 0.440 | ( | 0.198 | - | 0.974 | ) |
| CRP- |  | 18 | 10 | 19 | Homozygous | TT vs. CC | 0.437 | 0.700 | ( | 0.285 | - | 1.721 | ) |
|  |  | 44% | 23% | 53% | Heterozygous | TT vs. TC | 0.038* | 2.661 | ( | 1.043 | - | 6.790 | ) |
|  |  |  |  |  |  |  |  |  |  |  |  |  |  |
| rs4553808 | 203866221 | AA | AG | GG | Additive | AA vs.AG vs. GG | 0.704 | NA |  |  |  |  |  |
| CRP+ | CTLA4 | 61 | 11 | 2 | Dominant | AA vs. AG+GG | 0.629 | 1.279 | ( | 0.471 | - | 3.474 | ) |
|  |  | 59% | 69% | 50% | Recessive | AA+AG vs. GG | 1.000 | 0.653 | ( | 0.089 | - | 4.795 |  |
| CRP- |  | 42 | 5 | 2 | Homozygous | AA vs. GG | 1.000 | 0.689 | ( | 0.093 | - | 5.082 |  |
|  |  | 41% | 31% | 50% | Heterozygous | AA vs. AG | 0.468 | 1.515 | ( | 0.490 | - | 4.679 | ) |
|  |  |  |  |  |  |  |  |  |  |  |  |  |  |
| rs11571316 | 203866366 | GG | AG | AA | Additive | GG vs. AG vs. AA | 0.019* | NA |  |  |  |  |  |
| CRP+ | CTLA4 | 61 | 9 | 4 | Dominant | GG vs. AG+AA | 0.009* | 0.336 | ( | 0.147 | - | 0.772 | ) |
|  |  | 67% | 36% | 57% | Recessive | GG+AG vs. AA | 1.000 | 0.876 | ( | 0.187 | - | 4.097 | ) |
| CRP- |  | 30 | 16 | 3 | Homozygous | GG vs. AA | 0.685 | 0.656 | ( | 0.138 | - | 3.119 | ) |
|  |  | 33% | 64% | 43% | Heterozygous | GG vs. AG | 0.005* | 0.277 | ( | 0.110 | - | 0.699 | ) |
|  |  |  |  |  |  |  |  |  |  |  |  |  |  |
| rs62182595 | 203866465 | GG | AG | AA | Additive | GG vs. AG vs. AA | 0.619 | NA |  |  |  |  |  |
| CRP+ | CTLA4 | 60 | 12 | 2 | Dominant | GG vs. AG+AA | 0.535 | 1.367 | ( | 0.508 | - | 3.679 | ) |
|  |  | 59% | 71% | 50% | Recessive | GG+AG vs. AA | 0.646 | 0.639 | ( | 0.087 | - | 4.695 | ) |
| CRP- |  | 41 | 5 | 2 | Homozygous | GG vs. AA | 1.000 | 0.683 | ( | 0.092 | - | 5.048 | ) |
|  |  | 41% | 29% | 50% | Heterozygous | GG vs. AG | 0.382 | 1.640 | ( | 0.537 | - | 5.008 | ) |
|  |  |  |  |  |  |  |  |  |  |  |  |  |  |
| rs16840252 | 203866796 | CC | CT | TT | Additive | CC vs. CT vs. TT | 0.872 | NA |  |  |  |  |  |
| CRP+ | CTLA4 | 63 | 9 | 2 | Dominant | CC vs. CT+TT | 0.929 | 1.048 | ( | 0.376 | - | 2.920 | ) |
|  |  | 60% | 64% | 50% | Recessive | CC+CT vs. TT | 1.000 | 0.653 | ( | 0.089 | - | 4.795 |  |
| CRP- |  | 42 | 5 | 2 | Homozygous | CC vs. TT | 1.000 | 0.667 | ( | 0.090 | - | 4.919 |  |
|  |  | 40% | 36% | 50% | Heterozygous | CC vs. CT | 0.758 | 1.200 | ( | 0.376 | - | 3.831 | ) |
|  |  |  |  |  |  |  |  |  |  |  |  |  |  |
| rs5742909 | 203867624 | CC | CT | TT | Additive | CC vs. CT vs. TT | 0.787 | NA |  |  |  |  |  |
| CRP+ | CTLA4 | 56 | 16 | 3 | Dominant | CC vs. CT+TT | 0.526 | 1.323 | ( | 0.555 | - | 3.152 | ) |
|  |  | 59% | 67% | 60% | Recessive | CC+CT vs. TT | 1.000 | 0.979 | ( | 0.158 | - | 6.083 |  |
| CRP- |  | 39 | 8 | 2 | Homozygous | CC vs. TT | 1.000 | 1.045 | ( | 0.167 | - | 6.547 |  |
|  |  | 41% | 33% | 40% | Heterozygous | CC vs. CT | 0.489 | 1.393 | ( | 0.543 | - | 3.573 | ) |
|  |  |  |  |  |  |  |  |  |  |  |  |  |  |
| rs231775 | 203867991 | GG | AG | AA | Additive | GG vs. AG vs. AA | 0.275 | NA |  |  |  |  |  |
| CRP+ | CTLA4 | 39 | 25 | 8 | Dominant | GG vs. AG+AA | 0.244 | 0.635 | ( | 0.295 | - | 1.367 | ) |
|  |  | 68% | 63% | 47% | Recessive | GG+AG vs. AA | 0.136 | 0.458 | ( | 0.162 | - | 1.298 | ) |
| CRP- |  | 18 | 15 | 9 | Homozygous | GG vs. AA | 0.108 | 0.410 | ( | 0.136 | - | 1.237 | ) |
|  |  | 32% | 37% | 53% | Heterozygous | GG vs. AG | 0.545 | 0.769 | ( | 0.329 | - | 1.799 | ) |
|  |  |  |  |  |  |  |  |  |  |  |  |  |  |
| rs3087243 | 203874196 | GG | AG | AA | Additive | GG vs. AG vs. AA | 0.013* | NA |  |  |  |  |  |
| CRP+ | CTLA4 | 58 | 13 | 4 | Dominant | GG vs. AG+AA | 0.005* | 0.331 | ( | 0.152 | - | 0.722 | ) |
|  |  | 69% | 39% | 57% | Recessive | GG+AG vs. AA | 1.000 | 0.864 | ( | 0.185 | - | 4.038 | ) |
| CRP- |  | 26 | 20 | 3 | Homozygous | GG vs. AA | 0.675 | 0.598 | ( | 0.125 | - | 2.864 | ) |
|  |  | 31% | 61% | 43% | Heterozygous | GG vs. AG | 0.003* | 0.291 | ( | 0.126 | - | 0.673 | ) |
|  |  |  |  |  |  |  |  |  |  |  |  |  |  |
| rs11571319 | 203874215 | GG | AG | AA | Additive | GG vs. GA vs. AA | 0.698 | NA |  |  |  |  |  |
| CRP+ | CTLA4 | 53 | 12 | 10 | Dominant | GG vs. GA+AA | 0.396 | 1.434 | ( | 0.622 | - | 3.305 | ) |
|  |  | 58% | 67% | 67% | Recessive | GG+GA vs. AA | 0.601 | 1.354 | ( | 0.433 | - | 4.232 | ) |
| CRP- |  | 38 | 6 | 5 | Homozygous | GG vs. AA | 0.538 | 1.434 | ( | 0.453 | - | 4.536 | ) |
|  |  | 42% | 33% | 33% | Heterozygous | GG vs. GA | 0.506 | 1.434 | ( | 0.494 | - | 4.159 | ) |
|  |  |  |  |  |  |  |  |  |  |  |  |  |  |
| rs10204525 | 241850169 | TT | CT | CC | Additive | TT vs. CT vs.CC | 0.419 | NA |  |  |  |  |  |
| CRP+ | PDCD1 | 34 | 38 | 3 | Dominant | TT vs. CT+CC | 0.199 | 1.608 | ( | 0.778 | - | 3.322 | ) |
|  |  | 55% | 67% | 60% | Recessive | TT+CT vs. CC | 1.000 | 0.979 | ( | 0.158 | - | 6.083 | ) |
| CRP- |  | 28 | 19 | 2 | Homozygous | TT vs. CC | 1.000 | 1.235 | ( | 0.193 | - | 7.918 | ) |
|  |  | 45% | 33% | 40% | Heterozygous | TT vs. CT | 0.187 | 1.647 | ( | 0.783 | - | 3.466 | ) |
|  |  |  |  |  |  |  |  |  |  |  |  |  |  |
| rs56029561 | 241850737 | DEL | DEL/CAG | CAG | Additive | DEL vs. DEL/CAG vs.CAG | 0.525 | 1.792 | ( | 0.450 | - | 7.136 | ) |
| CRP+ | PDCD1 | 64 | 8 | 0 | Dominant | DEL vs.DEL/CAG+CAG | 0.525 | 1.792 | ( | 0.450 | - | 7.136 | ) |
|  |  | 60% | 73% | 0% | Recessive | DEL+DEL/CAG vs.CAG | NA | NA |  |  |  |  |  |
| CRP- |  | 43 | 3 | 0 | Homozygous | DEL vs.CAG | NA | NA |  |  |  |  |  |
|  |  | 40% | 27% | 0% | Heterozygous | DEL vs.DEL/CAG | 0.525 | 1.792 | ( | 0.450 | - | 7.136 | ) |
|  |  |  |  |  |  |  |  |  |  |  |  |  |  |
| rs2227981 | 241851121 | GG | AG | AA | Additive | GG vs. AG vs. AA | 0.049* | NA |  |  |  |  |  |
| CRP+ | PDCD1 | 32 | 28 | 7 | Dominant | GG vs. AG+AA | 0.027* | 2.422 | ( | 1.096 | - | 5.350 | ) |
|  |  | 51% | 76% | 58% | Recessive | GG+AG vs. AA | 1.000 | 0.933 | ( | 0.277 | - | 3.147 | ) |
| CRP- |  | 31 | 9 | 5 | Homozygous | GG vs. AA | 0.632 | 1.356 | ( | 0.389 | - | 4.731 | ) |
|  |  | 49% | 24% | 42% | Heterozygous | GG vs. AG | 0.014* | 3.014 | ( | 1.227 | - | 7.405 | ) |
|  |  |  |  |  |  |  |  |  |  |  |  |  |  |
| rs2227982 | 241851281 | AA | AG | GG | Additive | AA vs.AG vs. GG | 0.364 | NA |  |  |  |  |  |
| CRP+ | PDCD1 | 18 | 30 | 20 | Dominant | AA vs. AG+GG | 0.266 | 1.574 | ( | 0.705 | - | 3.512 | ) |
|  |  | 51% | 59% | 69% | Recessive | AA+AG vs. GG | 0.213 | 1.759 | ( | 0.719 | - | 4.303 | ) |
| CRP- |  | 17 | 21 | 9 | Homozygous | AA vs. GG | 0.155 | 2.099 | ( | 0.750 | - | 5.871 | ) |
|  |  | 49% | 41% | 31% | Heterozygous | AA vs. AG | 0.498 | 1.349 | ( | 0.567 | - | 3.208 | ) |
|  |  |  |  |  |  |  |  |  |  |  |  |  |  |
| rs6705653 | 241851407 | CC | CT | TT | Additive | CC vs. CT vs. TT | 0.070 | NA |  |  |  |  |  |
| CRP+ | PDCD1 | 34 | 28 | 8 | Dominant | CC vs. CT+TT | 0.030* | 2.329 | ( | 1.079 | - | 5.029 | ) |
|  |  | 51% | 74% | 62% | Recessive | CC+CT vs. TT | 0.863 | 1.110 | ( | 0.340 | - | 3.623 | ) |
| CRP- |  | 33 | 10 | 5 | Homozygous | CC vs. TT | 0.476 | 1.553 | ( | 0.460 | - | 5.237 | ) |
|  |  | 49% | 26% | 38% | Heterozygous | CC vs. CT | 0.022* | 2.718 | ( | 1.143 | - | 6.464 | ) |
|  |  |  |  |  |  |  |  |  |  |  |  |  |  |
| rs41386349 | 241851697 | GG | AG | AA | Additive | GG vs. AG vs. AA | 0.358 | NA |  |  |  |  |  |
| CRP+ | PDCD1 | 39 | 24 | 5 | Dominant | GG vs. AG+AA | 0.216 | 1.636 | ( | 0.752 | - | 3.557 | ) |
|  |  | 54% | 69% | 56% | Recessive | GG+AG vs. AA | 1.000 | 0.873 | ( | 0.222 | - | 3.436 | ) |
| CRP- |  | 33 | 11 | 4 | Homozygous | GG vs. AA | 1.000 | 1.058 | ( | 0.262 | - | 4.264 | ) |
|  |  | 46% | 31% | 44% | Heterozygous | GG vs. AG | 0.155 | 1.846 | ( | 0.788 | - | 4.324 | ) |
|  |  |  |  |  |  |  |  |  |  |  |  |  |  |
| rs36084323 | 241859444 | TT | CT | CC | Additive | TT vs. CT vs.CC | 0.184 | NA |  |  |  |  |  |
| CRP+ |  | 15 | 40 | 20 | Dominant | TT vs. CT+CC | 0.068 | 2.125 | ( | 0.940 | - | 4.806 | ) |
|  |  | 47% | 65% | 67% | Recessive | TT+CT vs. CC | 0.426 | 1.418 | ( | 0.598 | - | 3.361 | ) |
| CRP- |  | 17 | 22 | 10 | Homozygous | TT vs. CC | 0.116 | 2.267 | ( | 0.810 | - | 6.341 | ) |
|  |  | 53% | 35% | 33% | Heterozygous | TT vs. CT | 0.100 | 2.061 | ( | 0.865 | - | 4.907 | ) |
|  |  |  |  |  |  |  |  |  |  |  |  |  |  |
| rs5839828 | 241859601 | DEL | DEL/G | GG | Additive | DEL vs. DEL/G vs.GG | 0.175 | NA |  |  |  |  |  |
| CRP+ |  | 22 | 40 | 13 | Dominant | DEL vs. DEL/G+GG | 0.077 | 1.963 | ( | 0.926 | - | 4.160 | ) |
|  |  | 50% | 65% | 72% | Recessive | DEL+DEL/G vs.GG | 0.271 | 1.845 | ( | 0.613 | - | 5.551 | ) |
| CRP- |  | 22 | 22 | 5 | Homozygous | DEL vs.GG | 0.109 | 2.600 | ( | 0.792 | - | 8.535 | ) |
|  |  | 50% | 35% | 28% | Heterozygous | DEL vs. DEL/G | 0.135 | 1.818 | ( | 0.827 | - | 3.995 | ) |
| CRP: C-reactive protein; Additive: AA vs. Aa vs. aa; Dominant: AA vs. Aa+aa; Recessive: AA+Aa vs. aa; Homozygous: AA vs. aa; Heterozygous: AA vs. Aa, where the frequency of A-allele is major in the population, and a-allele is minor. *:<0.05; NA: not applicable.*: P<0.05; NA: not applicable | | | | | | | | | | | | | |
