# Supplementary material for: Exploration of the association between the single-nucleotide polymorphism of co-stimulatory system and rheumatoid arthritis
Source: Front Immunol. 2023 Jun 29;14:1123832. doi: 10.3389/fimmu.2023.1123832 (PMC10344454; doi:10.3389/fimmu.2023.1123832)
Supplement: Supplementary file 2 [file DataSheet_2.docx]

Exploration of the association between the single nucleotide polymorphism of co-stimulatory system and rheumatoid arthritis

**local_identifier NCBI_subsnp#**

rs181758110_G_A 2137544437

rs45454293 5982134873

rs1234314 5982134874

rs1879877 5982134875

rs3181096 5982134876

rs3181097 5982134877

rs3181098 5982134878

rs28718975 5982134879

rs28688913 5982134880

rs28541784 5982134881

rs201801072 5982134882

rs200353921 5982134883

rs56228674 5982134884

rs1290180288 5982134885

rs3116496 5982134886

rs11571315 5982134887

rs733618 5982134888

rs4553808 5982134889

rs11571316 5982134890

rs62182595 5982134891

rs16840252 5982134892

rs945677329 5982134893

rs5742909 5982134894

rs231775 5982134895

rs56217811 5982134896

rs980967681 5982134897

rs55696217 5982134898

rs3087243 5982134899

rs11571319 5982134900

rs10204525 5982134901

rs56029561 5982134902

rs2227981 5982134903

rs2227982 5982134904

rs6705653 5982134905

rs41386349 5982134906

rs36084323 5982134907

rs5839828 5982134908
